# Contrasting Ultra-Low Frequency Raman and Infrared Modes in Emerging Metal Halides for Photovoltaics

**DOI:** 10.1021/acsenergylett.4c01473

**Published:** 2024-07-29

**Authors:** Vincent J.-Y. Lim, Marcello Righetto, Siyu Yan, Jay B. Patel, Thomas Siday, Benjamin Putland, Kyle M. McCall, Maximilian T. Sirtl, Yuliia Kominko, Jiali Peng, Qianqian Lin, Thomas Bein, Maksym Kovalenko, Henry J. Snaith, Michael B. Johnston, Laura M. Herz

**Affiliations:** † Department of Physics, Clarendon Laboratory, University of Oxford, Parks Road, Oxford OX1 3PU, United Kingdom; ‡ Department of Physics, King’s College London, London WC2R 2LS, United Kingdom; § Department of Chemistry and Applied Biosciences, Institute of Inorganic Chemistry, ETH Zürich, Zürich 8093, Switzerland; ∥ Empa-Swiss Federal Laboratories for Materials Science and Technology, Dübendorf 8600, Switzerland; ⊥ Department of Chemistry and Center for NanoScience (CeNS), University of Munich (LMU), Butenandtstr. 11, 81377 Munich, Germany; # Key Lab of Artificial Micro- and Nano-Structures of Ministry of Education of China, School of Physics and Technology, Wuhan University, Wuhan 430072, Hubei, China; ∇ Institute for Advanced Study, Technical University of Munich, Lichtenbergstrasse 2a, D-85748 Garching, Germany

## Abstract

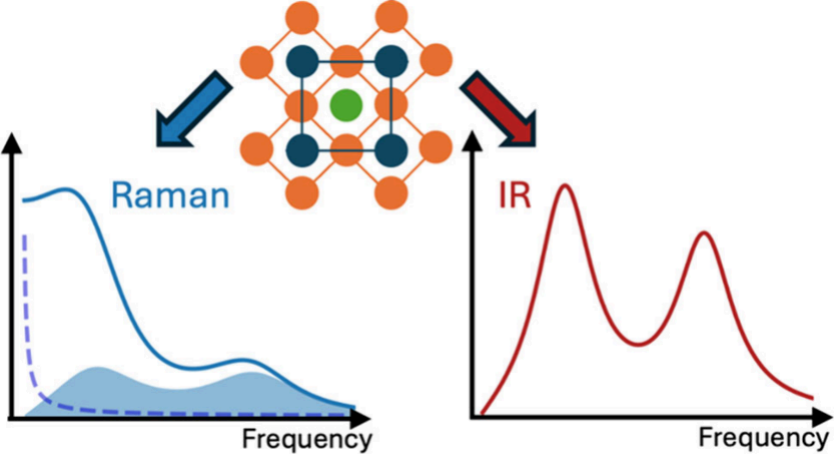

Lattice dynamics are critical to photovoltaic material performance, governing dynamic disorder, hot-carrier cooling, charge-carrier recombination, and transport. Soft metal-halide perovskites exhibit particularly intriguing dynamics, with Raman spectra exhibiting an unusually broad low-frequency response whose origin is still much debated. Here, we utilize ultra-low frequency Raman and infrared terahertz time-domain spectroscopies to provide a systematic examination of the vibrational response for a wide range of metal-halide semiconductors: FAPbI_3_, MAPbI_*x*_Br_3–*x*_, CsPbBr_3_, PbI_2_, Cs_2_AgBiBr_6_, Cu_2_AgBiI_6_, and AgI. We rule out extrinsic defects, octahedral tilting, cation lone pairs, and “liquid-like” Boson peaks as causes of the debated central Raman peak. Instead, we propose that the central Raman response results from an interplay of the significant broadening of Raman-active, low-energy phonon modes that are strongly amplified by a population component from Bose–Einstein statistics toward low frequency. These findings elucidate the complexities of light interactions with low-energy lattice vibrations in soft metal-halide semiconductors emerging for photovoltaic applications.

Lattice vibrations influence a plethora of fundamental material properties, ranging from dielectric and elastic responses to thermal and electronic conductivities.^1^ For semiconductors, coupling of phonons to charge carriers is critical to several photophysical processes–hot carrier cooling,^2,3^ dynamic disorder,^4^ and charge-carrier recombination^5^ – and most importantly imposes fundamental limits on charge-carrier transport.^6^ Metal-halide perovskites (MHPs) have attracted much attention for their excellent charge-carrier transport properties and their impressive potential in photovoltaic applications.^7^ Electron–phonon coupling in MHPs has been the subject of intense debate, and has often been proposed as the origin of their exceptional properties.^8,9^ Phonon frequencies in MHPs are lower compared to those in conventional inorganic semiconductors, as a result of heavier ions (e.g., Pb^2+^, I^–^) and mixed covalent-ionic bonds leading to a “soft” lattice.^9^ These low phonon frequencies have been stipulated to yield defect tolerance,^10^ anharmonicity of phonons^11^ and even a “liquid-like” nature of the material.^12,13^ In addition, charge-carrier properties are shown to be significantly influenced by structural fluctuations in MHPs, especially those induced by low-frequency vibrations.^8,14,15^

Understanding the role of the vibrational structure in determining the properties of MHPs is thus also crucial more generally for the development of a new family of high-performance semiconductors. Despite their ideal optoelectronic properties, MHPs can be affected by toxicity and structural, thermal and chemical instabilities.^16^ As a result, alternative metal-halide compositions and related semiconductors, often referred to as “perovskite-inspired” materials, are currently being discovered and explored for next-generation photovoltaic devices and other applications.^17^ Therefore, identifying the vibrational fingerprints of a promising emergent semiconductor is a fundamental step in the quest for developing such next-generation materials.

Raman spectroscopy in the THz region, also known as ultra-low frequency (ULF) Raman, is a well-established approach for the study of lattice dynamics of MHPs. It has been widely reported that MHPs exhibit a broad Raman response in this region, known as the “central Raman peak,”^18−20^ that increases in magnitude toward the elastically scattered (zero-frequency) light peak. Despite several studies on the low-frequency Raman response of MHPs, a consensus on the origin of this phenomenon has not yet been reached. There have been some initial assignments of this phenomenon to the “liquid-like” nature of the perovskite lattice^12,13,21^ causing local polar fluctuations,^18^ temperature-activated A-cation rotation,^22^ or octahedral tilting from cation lone pairs.^23^ IR and Raman spectroscopies can provide access to complementary information on the vibrational structure of semiconductors and a detailed comparison can be of unique value to a full understanding of the vibrational properties of MHPs. Crucially, the central Raman response visible in Raman spectra has not been reported in IR spectra,^24,25^ for reasons unexamined to date. However, the different nature of these responses–Raman is a light-scattering measurement, while IR spectroscopy measures light absorption–requires a careful comparison that considers the different transition matrices and the selection rules associated with these two techniques.

In this Letter, we report a systematic investigation of the low-frequency vibrational spectra recorded for a range of metal-halide semiconductors with relevance to photovoltaic applications. Our work elucidates the origin of the low-frequency vibrational response from these materials through a careful combined analysis of Raman and IR spectra. By studying a wide range of metal-halide compositions–in both thin film and single crystal form–and leveraging combined IR and Raman information, we are able to examine the origin of the central Raman response, and can rule out several potential explanations, including extrinsic defects, a double-well instability that may arise from octahedral tilting and cation lone pairs, and a boson peak that may be associated with a soft, “liquid-like” nature of the lattice. We suggest that instead, differences in the decay channels for Raman- and IR-active phonons, as well as a thermal population factor affecting solely the Raman intensity spectrum, are responsible for the peculiar low-frequency Raman response observed in many of these semiconductors.

To investigate the central Raman response, we employed ultra-low frequency (ULF) (>10 cm^–1^, ∼ 0.3THz) Raman and THz time-domain spectroscopy (THz-TDS), ideal techniques for probing Raman- and IR-active phonon modes in this range. For ULF Raman, a continuous wave (CW) pump laser with 900 nm wavelength was used, corresponding to photon energies below the band gap energy for all materials investigated, to avoid degradation from photoexcitation and contributions from resonant Raman conditions which may skew the intensities of certain modes.^26^ For THz-TDS, the transmission of THz-frequency pulses through thin films deposited on z-cut quartz was measured using electro-optic detection in the time domain, and the transients were Fourier transformed to yield the THz spectrum. Full descriptions of the spectroscopic techniques are given in Supporting Information Section 1. Here, we investigated a range of metal halide semiconductors currently being explored for photovoltaic and optoelectronic applications, including the lead-halide perovskites MAPbI_3_, FAPbI_3_, MAPbBr_1.5_I_1.5_, MAPbBr_3_, CsPbBr_3_ and their precursor PbI_2_, and the emerging metal halides Cs_2_AgBiBr_6_ and Cu_2_AgBiI_6_, as well as AgI for comparison. We note that in the literature, single crystals have been preferred for Raman investigations owing to their higher Raman scattering intensity and possibility for polarization-dependent measurements.^18,20,22,23,27,28^ However, to obtain an analysis more relevant to photovoltaic devices, we here explored thin films deposited on z-cut quartz, while also demonstrating comparison with single crystals for a subset of the materials investigated–fabrication details can be found in Supporting Information Section 2.

We examine the ULF Raman spectra of the various metal-halide semiconductor thin films to probe for the presence of the central Raman response, as shown in Figure 1. All the MHPs (MAPbI_3_, FAPbI_3_, MAPbBr_1.5_I_1.5_, MAPbBr_3_, CsPbBr_3_) exhibit such a central Raman peak, as do Cu_2_AgBiI_6_ and AgI, while PbI_2_ shows only a weak low-frequency response in that region. Cs_2_AgBiBr_6_ does not exhibit any central Raman peak, in agreement with reports by Cohen et al.^27^ For clarity, a zoomed-in plot of the Raman spectra of AgI and Cs_2_AgBiBr_6_ can be found in Figure S2 of the Supporting Information. It is worth noting that from ideal point-group analysis, materials with perfect cubic perovskite structure should not possess any Raman-active phonons.^29^ Therefore, for MHPs to exhibit a strong low-frequency Raman response, some disorder or anharmonicity needs to be present.^30,31^ Alternatively, this low-frequency response could be caused by multiphonon processes,^32,33^ but no higher harmonics of this response appear visible in the Raman spectra. Moreover, we note that sharp features in the Raman response of the metal-halide thin films are superimposed with the broad central Raman response, similar in appearance to optical phonon modes apparent in IR spectra discussed below. This coexistence of a broad feature with more sharply defined phonon peaks suggests that not just one type of lattice dynamic causes the low-frequency Raman response of MHPs.

**Figure 1:**
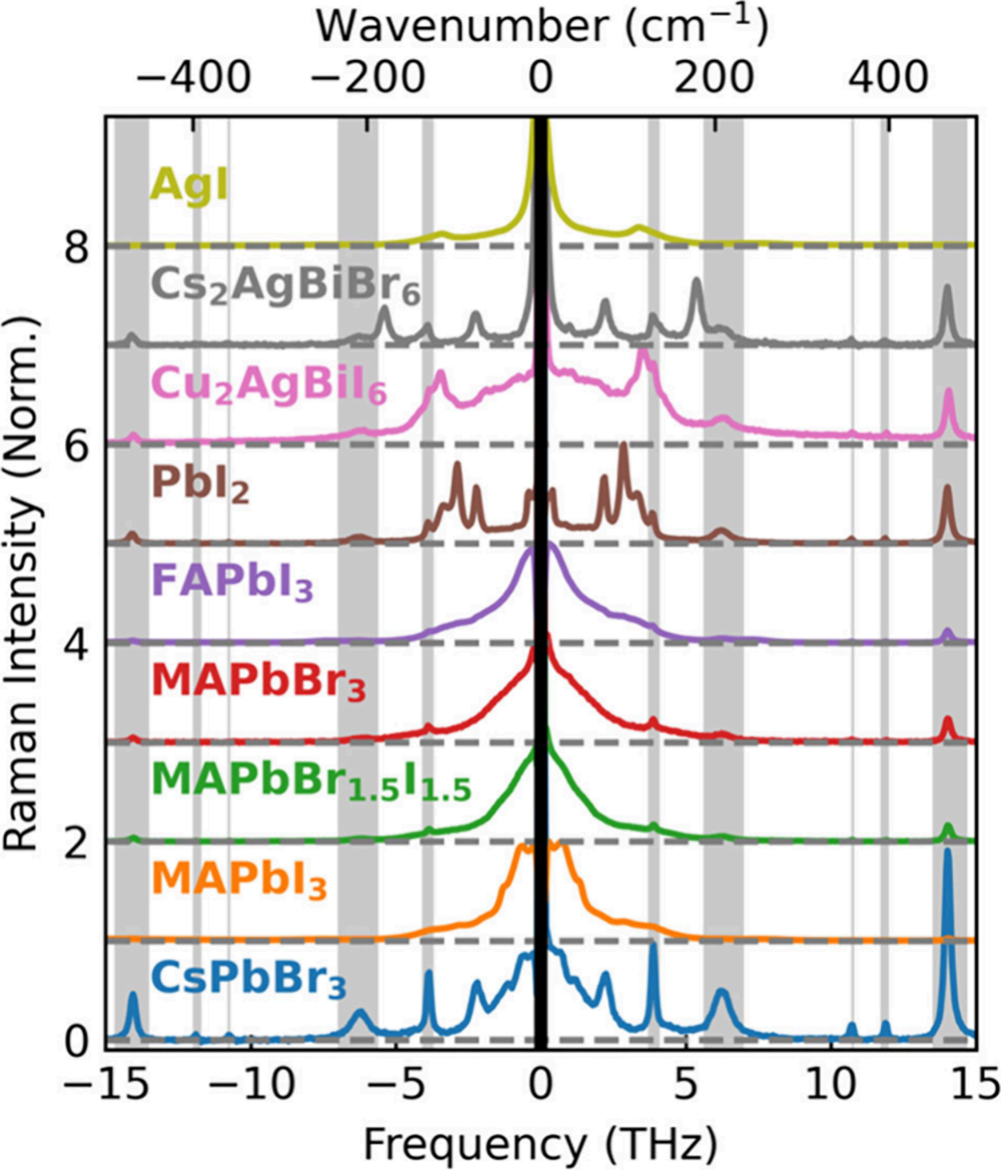
Raman spectra of a set of thin-film metal halides, as indicated. The central Rayleigh scattering peak is hidden with black, and the phonon responses of the z-cut quartz substrate are indicated with gray throughout the spectra. A CW laser with wavelength of 900 nm was used as the light source, and the Raman signal was collected in a backscattering geometry. Rayleigh scatter was suppressed with volume-Bragg notch filters. Experimental details can be found in Supporting Information section 1. A table of phonon mode frequencies from fitting can be found in Table 1 in Supporting Information section 5.

We start by discussing and assessing–based on the Raman data reported in Figure 1 and prior studies–the main factors proposed in the literature as origins of the central Raman response for MHPs, in the following order: the presence of a double-well potential from A-cation lone pairs or octahedral tilting, extrinsic disorder from defects and impurities, and a boson peak response arising from a soft “liquid-like” nature of the structure.

The presence of a double potential well in the lead halide octahedral cage [PbX_6_]^4–^ and the dynamic instability associated with this lattice potential has been intensely discussed in the literature for MHPs.^30,34−38^ Structural distortion in the perovskite lattice associated with this potential could explain the presence of the low-frequency Raman response, as a result of deviation from the perfect cubic structure. Recently, the double-potential-well model has also been invoked to account for the anomalous temperature dependence of the Raman intensity of some oxide and metal-halide perovskite materials.^30^ Here, we note that the presence of a double-well potential in MHPs can be intimately connected with a stereochemically expressed 6s^2^ lone pair from the Pb^2+^ cation.^36−38^ Interestingly, no similar double-well potential is expected for AgI and for CsSrBr_3_,^39^ given the electronic configuration of the Ag^+^ and Sr^2+^ cations ([Kr]4d^10^ 5s^0^) and ([Kr]5s^0^) lack such *ns*(2) lone pairs. However, these materials also exhibit a broad low-frequency Raman response (see Figure 1 for AgI and ref (39) for CsSrBr_3_). As recently shown, CsSrBr_3_ shows close structural similarity with CsPbBr_3_, but unlike the latter exhibits no lone pair, yet both exhibit a broad central Raman response in their high-temperature cubic phase.^39^ Overall, we therefore rule out the stereochemical activity of heavy cation lone pairs as the primary factor in determining the presence of a central Raman response in these metal halide semiconductors.

Octahedral tilting has been proposed as an alternative possible cause of instabilities related to double-well potentials in MHPs.^15,34,35^ This effect is closely related to the Goldschmidt tolerance factor and therefore the choice of A-cation; when A-cations are smaller than the optimal size for bonding with BX_3_, octahedral tilting may occur to compensate.^40−42^ Octahedral tilting has been associated with zone-boundary vibrational instabilities giving rise to anharmonic double-well potentials;^40−42^ therefore different A-cations are expected to cause octahedral tilting and double-well potentials to different extents. However, we observe that MHPs differing only in their A-cations (i.e., MA, FA and Cs in Figure 1, as well as MHy^20^ and 2D MHPs^43^ reported in the literature, with differing values of the Goldschmidt tolerance factor) exhibit a very similar central Raman response. We therefore suggest that octahedral tilting cannot play a major role in the appearance of a central Raman feature either.

Another possible source of disorder may arise from extrinsic factors related to processing conditions, i.e. defects and interstitials, chemical inhomogeneity,^45^ or crystallinity/grain boundaries. It has indeed been shown that defects and impurities can influence the low-frequency Raman response.^46,47^ A defect can distort a unit cell which can oscillate between unit cells with different symmetries,^46^ or an impurity can cause a strain field surrounding it, so both may in principle cause a central Raman response.^47^ We aim to examine the presence of such effects in MHPs by comparing Raman measurements on thin films with those of single crystals, for materials with the same nominal composition, but with distinctly different defect densities. For MHPs in particular defect densities have been reported to be of the order of ∼10^17^ cm^–3^ for thin films^48^ and ∼10^10^ cm^–3^ for single crystals,^48,49^ therefore we contrasted ULF Raman spectra measured both for thin films and single crystals of CsPbBr_3_ and MAPbI_3_ in Figure 2(a, b). We observe that both forms of the same material exhibit very similar responses in the low-frequency region of the Raman spectra, for both compositions. We therefore conclude that such extrinsic sources of disorder are unlikely to be related to the central Raman response in MHPs.

**Figure 2:**
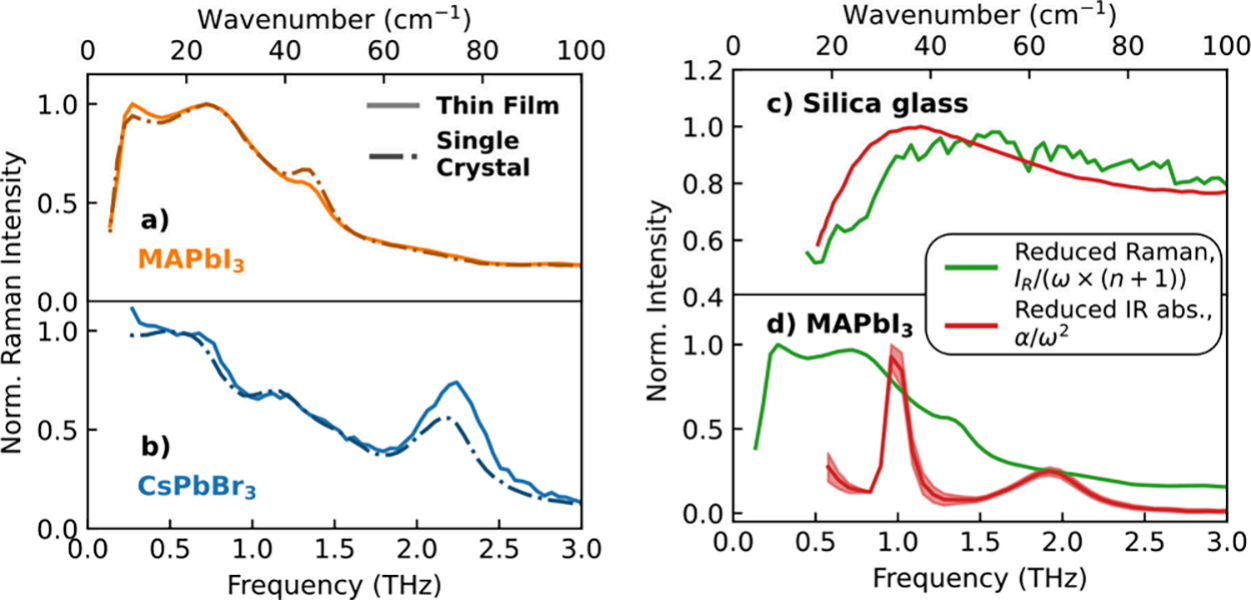
Normalized Raman spectra of thin films (solid lines) and single crystals (dash-dot lines) of (a) MAPbI_3_ and (b) CsPbBr_3_. (c) Reduced Raman (scheme 2) and reduced IR absorption spectra of amorphous silica glass, with the peak near 1 THz being the Boson peak arising from the liquid-like nature of amorphous silica glass. IR absorption data were taken from ref (44). (d) Normalized reduced Raman and reduced IR absorption spectra of MAPbI_3_ thin film on z-cut quartz. The reduced Raman spectrum is given by *I*_R_/(ω × (*n* + 1)), where *I*_R_ is the measured Raman intensity, and the reduced IR spectrum is given by α/ω^2^, where α is the measured THz IR spectrum (scheme 2). A laser wavelength of 900 nm was used for nonresonant Raman spectroscopy, and THz-TDS was employed for acquisition of the IR absorption spectrum, as detailed in Supporting Information Section 1. IR spectra for further metal-halide semiconductors are provided in Supporting Information Figure S3.

We further probe whether the central Raman peak may form as a result of a “liquid-like” nature that has been postulated for MHPs^12,13^ owing to their exceptionally soft lattice.^50^ An amorphous material may exhibit a low-frequency Raman response, known as the Boson peak,^51−56^ because if disorder within a material is sufficiently high, selection rules will be lifted since each mode will no longer be localized in momentum space and cannot be characterized by a single wavevector.^57,58^ Therefore, both optical and acoustic modes will contribute to the vibrational density of states (vDoS),^57,58^ giving rise to an excess vDoS adding onto the Debye vDoS (∝ω^2^).^54−56^ This phenomenon has been identified as the primary cause for the presence of a low-frequency Boson peak in amorphous materials such as silica glass.^44,56^ Crucially, the lifting of selection rules implies that this additional vDOS contributes to signal amplitudes in both Raman and IR spectra.^54^ However, to allow for clear observation of the Boson peak and for an accurate comparison between Raman and IR spectra, a spectral reduction must first be applied to the experimentally measured Raman and IR spectra to account for differences in the measurement techniques and yield a spectrum accurately describing the vDOS. The intensity of the Raman signal generated by harmonic oscillators is proportional to ω/(*n*+1), and for disordered materials with short spatial correlation length, the Raman susceptibility χ^″^ can thus be described by the relation:^54,57,58^
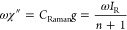
1where *C*_Raman_ is the Raman coupling coefficient, *g* is the vDoS, *I*_R_ is the Raman intensity (as measured), and *n* is the Bose–Einstein distribution. We label this reduction scheme as scheme 1. The IR absorption spectrum has a simpler form:^54^
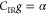
2where *C*_IR_ is the IR coupling factor, and α is the IR absorption spectrum (as measured). We note that *I*_R_, α, χ″, *C*_Raman_, *C*_IR_, *g*, and *n* all depend on the frequency ω of the transmitted or scattered photon. Importantly, after the appropriate spectral reduction is applied to the measured signals, the reduced IR and Raman spectra are both representative of the vDoS, apart from the application of symmetry-related selection rules that may differ between the two techniques. In order to more clearly observe the Boson peak on top of a Debye vDoS (∝ω^2^), we follow the standard literature approach to further reduce the IR and Raman intensities to α/ω^2^ and *I*/[ω(*n* + 1)], respectively, which are both proportional to *g*/ω^2^,^51−54^ and we label this as scheme 2.

There have been literature reports postulating a “liquid-like” nature of MHPs, based on large phonon dynamics and polaron formation.^12,13^ Such “liquid-like” nature was also postulated as a possible cause for the central Raman response.^18,22,23^ If MHPs are “liquid-like”, i.e. they have almost the response of an amorphous material, the Boson peak should be visible, and may indeed be the cause of the central Raman response.^54,59^ We thus examine this question by comparing reduced Raman and IR spectra.^54,59^ To ensure our method is correctly implemented, we first examine silica glass, an established amorphous material. As Figure 2(c) shows, for silica glass, the reduced IR and Raman spectra are indeed very similar (IR spectrum taken from ref (44)) with the Boson peak visible at ∼1 THz, with minor deviation attributed to differences in coupling coefficients.^54,59^ In contrast, for all MHPs, we instead observe clear deviations between the reduced IR and Raman spectra (see Figure 2(d) for MAPbI_3_ and Figure S3 for other MHPs). We note that the deviations between the two types of reduced spectra cannot be attributed solely to differences in the coupling coefficients applying to Raman and IR signals. The clear differences between the broadness of the measured peaks, as well as their shape and position suggest more profound causes for the divergence between the Raman and IR responses. Overall, we thus conclude that the central Raman response in MHPs does not originate from the appearance of a Boson peak, or a “quasi-amorphous” nature of the material.

It is evident that the Raman and IR responses are very distinct in MHPs, although they should both operate based on the same vDoS, albeit with different selection rules. We continue to explore potential reasons for the discrepancy between Raman and IR spectra, focusing in particular on the differences in the central, low-frequency Raman response. To contrast the spectra appropriately, we use the reduction schemes for Raman and IR spectra discussed above, which have been shown to be effective for amorphous,^54,60^ as well as crystalline materials.^61−63^Figure 3 shows the reduced Raman and IR spectra for a range of metal halide semiconductors, but the spectral shapes are clearly very different. (We note that following convention, the reduction scheme here is scheme 1 and therefore different to the scheme 2 applied to data shown in Figure 2(c,d) in order to highlight the Boson peak.) Unreduced Raman and IR spectra of all the samples under consideration, along with fitting and extracted parameters, can be found in Supporting Information Section 5, Figures S5–19. Reduced Raman spectra shown in Figure 3(a) exhibit broad, almost linear, responses toward zero frequency, whereas IR absorption spectra of the same semiconductors in Figure 3(b) generally show well-defined phonon peaks with little signal toward zero frequency (AgI, Cs_2_AgBiBr_6_ and Cu_2_AgBiI_6_ do not present strongly IR-active phonons in this range). Some of the differences in Raman and IR spectra will clearly be caused by changes in coupling coefficients for specific modes. Determination of coupling coefficients for metal-halide semiconductors are still ongoing, either thorough experimental studies, i.e. neutron scattering and heat capacity measurements, or theoretical studies.^64−66^ Nevertheless, beyond such effects, it is evident from the data in Figure 3 that the broadening of the Raman and the IR responses differs significantly.

**Figure 3:**
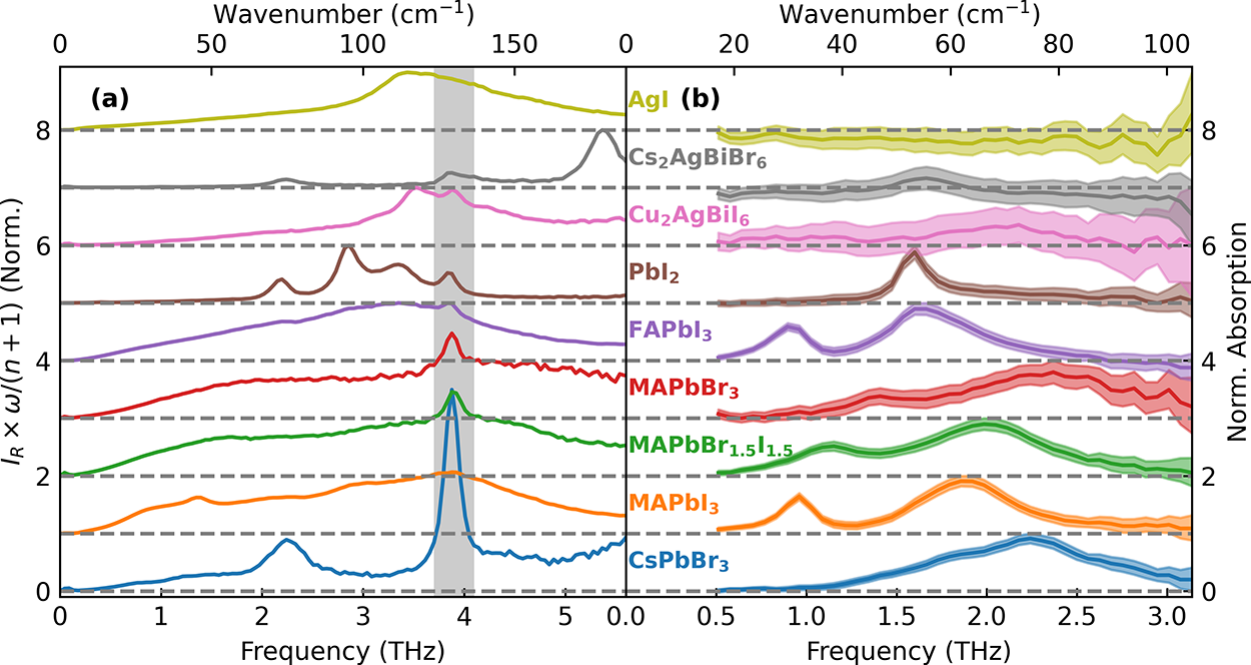
(a) Normalized reduced Raman spectra (scheme 1) of metal halides deposited as thin films on z-cut quartz. The phonon response from the quartz substrate is highlighted in gray. (b) Normalized IR absorption of the same metal halides (equivalent to the reduced Raman spectra, as can be seen from eqs 1 and 2), acquired with THz-TDS. A table of phonon mode frequencies from fitting can be found in Table 1 in Supporting Information section 5. A plot with the same frequency scale is presented in Figure S4 in the Supporting Information.

We proceed by exploring the hypothesis that the central Raman response is caused by the broadness of the specific phonon responses observable in Raman spectra covering the ultra-low frequency region, along with the Bose–Einstein population factor which significantly amplifies Raman signals toward zero frequencies. Both for MHPs^19,23^ and other materials,^67,68^ Raman spectra have been approximated with a series of damped harmonic oscillators (described by the Lorentz model) multiplied by the Bose–Einstein (*n*(ω) + 1) term, where *n*(ω) = 1/(*e*^*ℏω*/*k*_*b*_*T*^ – 1), and *ℏ*, *k*_*b*_, *T* are reduced Planck’s constant, Boltzmann constant and absolute temperature, respectively. Within the above formalism (eq 1), this is equivalent to the assumption that the Raman coupling coefficient is linear in frequency, i.e. *C*_*R*_ ∝ ω.^64−66^ We also adopt this approach, and employ the Lorentz damped harmonic oscillator model, which predicts a scattering cross-section per oscillator mode *i* as
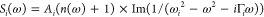
3where *A*_*i*_, *n*, ω_*i*_, and Γ_*i*_ are the amplitude, Bose–Einstein factor, harmonic oscillator frequency, and damping coefficient, respectively. We fitted the ULF Raman spectra (up to 3 THz) of MA-based MHPs, shown in Figure 1, to the sum of three phonon resonances with scattering cross sections described by eq 3, as shown in Supporting Information Section 5 (Figures S5–S7).^19^ The above Lorentz equation, but without the (*n*(ω)+1) Bose–Einstein term and with two phonon resonances, was also fitted to the IR absorption spectra, also shown in Supporting Information Section 5 (Figures S14–S16). To quantify the extracted broadening of these phonon modes, we calculate and show the ratio Γ/ω_0_ in Figure 4(a), which reveals that the lattice response observed in Raman spectra at ultra-low frequencies is significantly broader than that evident in IR spectra. This discrepancy may arise from the types of modes probed by the two techniques. As shown in various computational studies on lead halide perovskites, the ultra-low frequency range (<100 cm^–1^) hosts a large number of IR- and Raman-active modes.^28,69−73^ These have primarily been associated with different types of motions of the lead-halide sublattice, in agreement with the blueshift we observe in our Raman and IR spectra for the phonon frequencies of MA-based MHPs when the bromide composition increases, as shown in Figures S19 and S20. Thus, the significantly enhanced broadening of the ULF Raman modes compared to the IR modes in the same range may result from different Raman- and IR-coupling rules for different types of modes.

**Figure 4:**
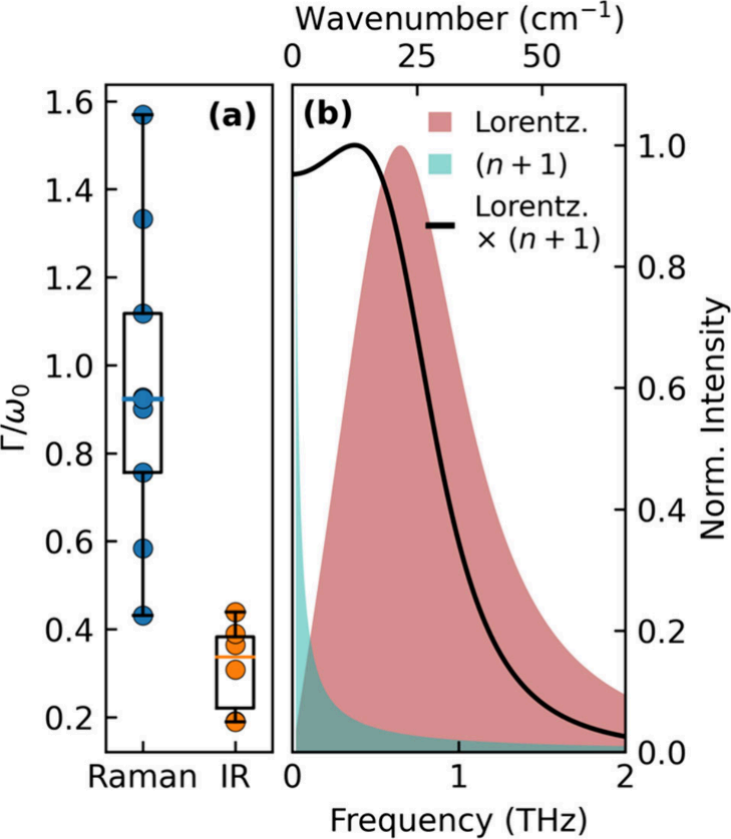
(a) Box plot showing the broadening, quantified by Γ/ω_0_, of lattice vibrations in the THz-frequency range determined for MA-based metal-halide perovskites (MAPbI_3_, MAPbI_1.5_Br_1.5_, MAPbBr_3_) from Raman or THz-TDS IR absorption measurements, as detailed in Supporting Information section 1. (b) Illustration of a damped harmonic oscillator response (Lorentz model, eq 3), Bose–Einstein population factor (*n*(ω) + 1), where *n* (ω) = 1/(e^ℏω/*k*_b_*T*^ – 1), and the multiplication of the two, demonstrating the expected Raman response for a single low-frequency mode according to the damped harmonic oscillator model. Parameters used were ω_0_ = 0.8 THz, Γ = 1 THz, and *T* = 300 K.

In this context, it is important to note that while Raman can (if symmetry permits) probe both transverse optical (TO) and longitudinal optical (LO) phonons and modes,^28^ THz-TDS conducted at near-normal incidence only probes transverse optical phonons since the electric field of the THz wave oscillates perpendicularly to its direction of propagation,^24^ though IR measurements conducted at a significant angle can provide some access to IR-active LO phonons.^74^ From a very general perspective, differences in line widths observed in Raman and IR spectra may thus arise from variations in the decay channels operating for LO and TO phonons. LO and TO phonons share some similar decay mechanisms, i.e. decay to acoustic phonons,^75^ or through phonon–phonon scattering.^76^ However, their decay rates will differ, since LO and TO phonons will decay into a range of different acoustic phonons.^77^ LO phonons have also been reported to transform into one optical mode and one acoustic mode,^78,79^ and can additionally experience Fröhlich coupling^69,80^ to intrinsic charge carriers, which may shorten phonon lifetimes and thus broaden the Raman response further.

More specifically to lead-halide perovskites, accurate calculations of phonon mode frequencies have often been hindered by softness and structural flexibility of the lattice. While first-principles calculations agree that ULF modes in lead-iodide perovskites originate from Pb–I–Pb rocking and bending modes,^28,69−73^ they often suffer from significant shifts in calculated peak frequencies compared to experimental data,^28,73^ or do not capture^69^ the experimentally observed^28,73^ ultra-low frequency peaks near 10–20 cm^–1^. Such discrepancies may arise from lattice anharmonicity, which has been proposed to be substantial in metal halide perovskites.^35,81−83^ Moreover, the perfect cubic perovskite structure does not have any Raman-active modes,^29^ but anharmonicity can cause deviation from the perfect cubic structure, and therefore lift the Raman selection rules. Indeed, Figure 4(a) indicates that the phonon mode broadening Γ approaches the value of its frequency ω_0_ for Raman-active ULF soft modes in lead halide perovskites, a clear signature of anharmonic effects. The anharmonic low-frequency motions were investigated with molecular dynamics (MD) calculations for lead-halide perovskites that revealed lattice dynamics arising from head-to-head motion of A-cations in the cuboctahedral voids of the perovskite structure and octahedral distortions.^18^ Anharmonic polar rotation motions of hybrid A-cations have also been identified.^84^ Such MD simulations revealed particularly low (subpicosecond) phonon lifetimes associated with these ULF modes, that further shortened as frequencies were lowered from 3 to 1 THz.^85^ While further work is needed to assign and contrast the Raman and IR intensities of soft lattice modes from such MD calculations, such information may ultimately hold the key to understanding the differences in broadening observed for ULF Raman- and IR-active modes.

Overall, we therefore posit that the central Raman response of many metal halide perovskites results from an interplay of the significant broadening of Raman-active broad soft phonon modes in the low-frequency region, amplified by the (*n*+1) Bose–Einstein population factor, which becomes substantial at low frequencies (see Figure 4(b)). As a simple demonstration of these two effects, Figure 4(b) shows the response for a single oscillator mode according to the Lorentz model together with the Bose–Einstein population factor, along with the multiplication of the two. This should be comparable to the Raman response, with the assumption of *C*_*R*_ ∝ ω^64−66^ within the formalism in eq 1. The resulting function represented by the black line is clearly very comparable in shape to the central Raman response experimentally observed. In particular, we note that this simulated spectrum accurately reflects the peculiarity of ULF Raman spectra of lead-halide perovskites, i.e., the presence of both peak-like features and a seemingly continuous background heading to large amplitude toward lower frequencies. The broad central Raman response toward zero scattering frequencies is thus the result of the particularly low-energy lattice vibrations in these materials, which tail into the frequency-range where the population factor becomes significant. As such, it therefore does not reflect a continuum of vibrational frequencies, but rather the presence of individually defined but very broad modes. We also note that such broad low-frequency Raman response of MHPs has been shown to increase with temperature,^18,27,43^ agreeing well with our hypothesis. On the other hand, the IR response does not show such low-frequency response because of two combined effects: first, IR measurements are light absorption measurements, in which one IR photon is converted into a phonon, so do not depend on the initial population of vibrational modes. In contrast, Raman scattering requires interaction with an existing ground- or higher state phonon population, and therefore the Bose–Einstein population factor becomes prominent in Raman scattering. Second, the IR-active TO phonon modes exhibit far narrower line widths stemming from different decay mechanisms compared to those operating for the lowest-frequency, most anharmonic Raman-active modes. We thus conclude that the central Raman response results from the presence of significantly broadened low-energy Raman-active lattice modes whose anharmonic response is amplified by the Bose–Einstein population factor, and that the existence of these modes is the criterion for such central Raman response.

In conclusion, we examined the lattice responses in the low-frequency THz regime across a range of metal halide semiconductors, contrasting Raman- and IR-active vibrations. We employed ultra-low frequency Raman and THz-TDS IR spectroscopy, observing clear discrepancies in the spectra obtained. While Raman spectra exhibit a broad central response rising toward zero frequency, no such response is visible in the IR spectra, which only display well-defined resonance peaks. We systematically examined the cause behind the central Raman response, and the observed discrepancies between Raman and IR spectra. We excluded the following possibilities as the primary causes of the central Raman response: a Boson peak arising from a proposed “liquid-like” nature of these materials, dynamic disorder originating from A-cations, lone pairs, or octahedral tilting, and static disorder associated with extrinsic defects. Instead, we attribute the central Raman peak to a combination of particularly strongly broadened low-energy Raman-active modes whose response thus tails into the range where the Bose–Einstein factor governing phonon population becomes very significant. The IR response of these metal halide semiconductors, on the other hand, does not show an equivalent feature near zero frequencies because the line widths of the TO phonons governing IR absorption is narrower, and the Bose–Einstein population factor does not contribute. Overall, our findings reveal the complexities of light-matter interactions in soft metal-halide semiconductors governed by significant low-energy lattice dynamics. Understanding such lattice dynamics in soft semiconductors will help discover and engineer next-generation novel materials for photovoltaic applications.

## Supporting Information Available

Experimental details of spectroscopic techniques, sample fabrication details, comparison of Raman spectra for AgI and Cs_2_AgBiBr_6_ thin films, deviation between reduced Raman and IR spectra for a range of MHPs, and fitting of Raman and THz IR spectra (PDF)
