# Supplementary material for: Contrasting Ultra-Low Frequency Raman and Infrared Modes in Emerging Metal Halides for Photovoltaics
Source: ACS Energy Lett. 2024 Jul 29;9(8):4127–35. doi: 10.1021/acsenergylett.4c01473 (PMC11320646; doi:10.1021/acsenergylett.4c01473)
Supplement: Supplementary file 1 — nz4c01473_si_001.pdf [file nz4c01473_si_001.pdf]

Supporting Information: Contrasting Ultra-Low Frequency Raman and Infrared Modes in Emerging Metal Halides for Photovoltaics

Vincent J.-Y. Lim[1], Marcello Righetto[1], Siyu Yan[1], Jay B. Patel[2], Thomas Siday[1], Benjamin Putland[1], Kyle M. McCall[3,4], Maximilian T. Sirtl[5], Yuliia Kominko[3,4], Jiali Peng[6], Qianqian Lin[6], Thomas Bein[5], Maksym Kovalenko[3,4], Henry J. Snaith[1], Michael B. Johnston[1], Laura M. Herz[1,7,]*

[1] Department of Physics, Clarendon Laboratory, University of Oxford, Parks Road, Oxford, OX1 3PU, United Kingdom

[2] Department of Physics, King's College London, London, WC2R 2LS, United Kingdom

[3] Department of Chemistry and Applied Biosciences, Institute of Inorganic Chemistry, ETH Zürich, Zürich, 8093, Switzerland

[4] Empa-Swiss Federal Laboratories for Materials Science and Technology, Dübendorf, 8600, Switzerland

[5] Department of Chemistry and Center for NanoScience (CeNS), University of Munich (LMU), Butenandtstr. 11, 81377 Munich, Germany

[6] Key Lab of Artificial Micro- and Nano-Structures of Ministry of Education of China, School of Physics and Technology, Wuhan University, Wuhan 430072, Hubei, China

[7] Institute for Advanced Study, Technical University of Munich, Lichtenbergstrasse 2a, D-85748 Garching, Germany

Corresponding Author: laura.herz@physics.ox.ac.uk

1. Experimental details of spectroscopic techniques

1.1 Ultra-low-frequency (ULF) Raman spectroscopy

A home-built ULF Raman setup was employed to measure Raman spectra in the ULF region. A Spectra Physics 3900S Ti:Sapphire continuous-wave (CW) laser, pumped with a Millennia (Spectra-Physics) 532nm CW laser was used as the incident light. The central wavelength was set to 900nm, and two band pass filters were used to suppress spontaneous emission from the laser and further narrow its spectrum before the light was incident on the sample. The light was focused onto the sample and the scattered light collected in a back scatter geometry using a 0.5 NA microscope objective (Olympus LMPLFLN50x). The back-scattered light was collimated and incident on a beamsplitter, used to reflect a narrow (elastically scattered) wavelength band centred around 900nm, and to pass through the inelastically scattered light with wavelengths to either side of this central band. Two notch filters were used to further suppress the elastic (Rayleigh) scatter, followed by spatial filtering, and finally, wavelength dispersion of the Raman scatter with a Horiba iHR320 spectrometer using a 600 g/mm, 750nm central wavelength grating, and detection by a Symphony silicon CCD. The band pass filters, beamsplitter and notch filters were volume Bragg filters from OptiGrate,[1] fabricated for a central wavelength of 900nm. A background spectrum was acquired by measurement of a Raman spectrum without a sample in place but otherwise identical measurement conditions,

and was subtracted from the measured Raman spectra recorded in the presence of a sample. All Raman spectra were intensity corrected using a tungsten-filament reference lamp of known emissivity spectrum. Also, the intensity of Raman scattering is directly proportional to the fourth power of the frequency, which has been corrected for all Raman spectra.[2] The band pass and notch filters have FWHM linewidth of 5cm^{-1}. Therefore, we regard the lower limit of the accessible range as ~7cm^{-1}, but this can vary from sample to sample depending on the exact alignment for each sample, sample surface quality etc.

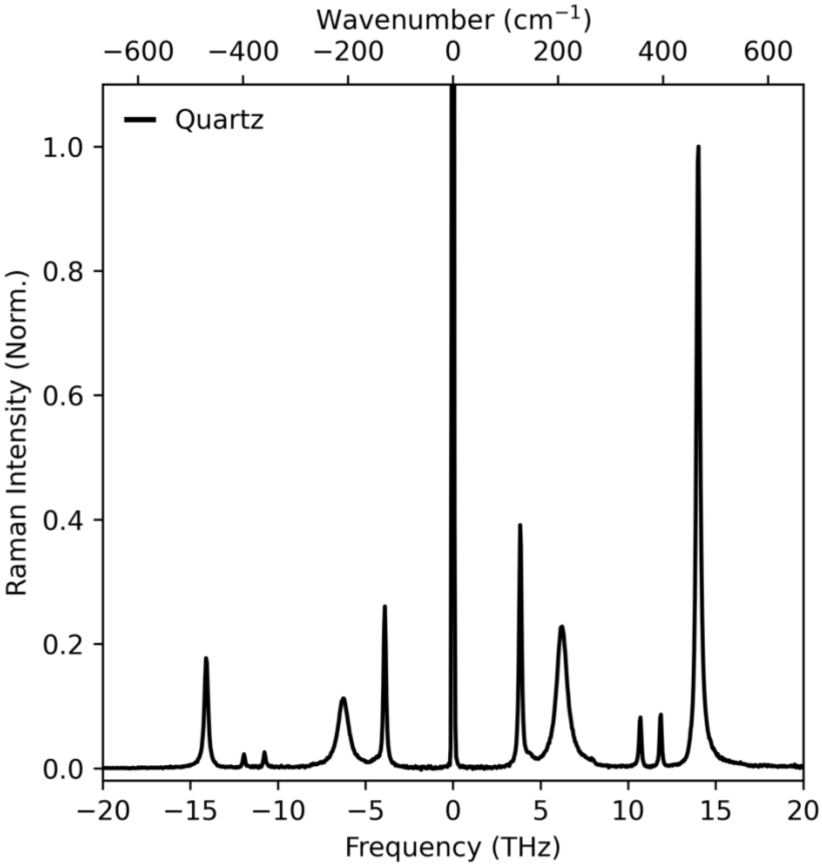

Figure S1 Normalised Raman intensity spectra of a z-cut quartz, the substrate used throughout this study. A CW laser with wavelength 900nm was used as the light source, and the Raman signal was collected in a back-scattering geometry. Rayleigh scatter was suppressed with volume-Bragg notch filters. Experimental details can be found in Supporting Information Section 1.1 above.

1.2 Terahertz time-domain spectroscopy (THz-TDS)

The experimental details for acquisition of THz-frequency IR absorption spectra have already been published in previous studies.[3–5] Briefly, an amplified Ti:Sapphire laser system was used for generation and detection of THz pulses, with characteristics ~ 35 fs pulse duration, 800 nm central wavelength, 5 kHz repetition rate (MaiTai – Ascend – Spitfire regenerative amplifier from Newport Spectra Physics). THz pulses were generated using a tri-layer spintronic emitter (2 nm tungsten, 1.8 nm $Co_{40}Fe_{40}B_{20}$, 2 nm platinum on a quartz substrate). These were focused onto the thin films deposited on z-cut quartz substrates using gold-coated off-axis parabolic mirrors, and then onto a 1 mm-thick (110)-ZnTe crystal for electro-optic sampling. The polarisation of the gate beam was detected with the combination of a quarter-wave plate, polarising beam splitter and a balanced photodiode. A home-made field-programmable gate-array-based board was used for data acquisition. Relative time delays were controlled using optical delay stages. We take ~0.5THz as the lower limit of our detection due to the lower frequency components not being able to be fully collected by OAPs and also not being focused tightly onto the detection crystal.

2 Sample fabrication details

2.1 $MAPbI_3$, $MAPbBr_{1.5}I_{1.5}$ and $MAPbBr_3$ thin films

$MAPbI_3$, $MAPbBr_{1.5}I_{1.5}$ and $MAPbBr_3$ thin films were prepared using the acetonitrile route as reported previously.[6,7] In brief, MAI (Greatcell), MABr (Greatcell), PbI_2 (TCI Chemicals 99.99%) and $PbBr_2$ (Thermo Scientific 98%) were weighed out and dissolved in a methylamine/acetonitrile (Merck Chemicals) solvent system to give a 0.5 M perovskite solution as described by Noel *et al*.[6] The solutions were then statically spincoated onto quartz substrates in a nitrogen filled drybox at 2000 rpm for 45 s. The films were then annealed for 90 minutes at 100 °C.

2.2 $MAPbI_3$ single crystals

The $MAPbI_3$ perovskite single crystals were prepared via inverse temperature crystallization.[8,9] Typically, 1.3 M $CH_3NH_3PbI_3$ precursors were prepared by adding 2.3 g lead iodide (PbI_2) and 0.8 g methylammonium iodide (MAI) into 3.85 mL γ–butyrolactone (GBL), heated at 90 °C for

2 hours with stirring. Then, the precursor solutions were filtered with syringe filters (0.22 μm pore size) and transferred to clean containers, which were kept on a stable hot-plate and heated at 130 °C for 3 hours. Crystals were formed on the bottom of the containers. Finally, the crystals were collected and dried at 60 °C in glovebox for 2 hours.

2.3 $FAPbI_3$ thin films

FAI and PbI_2 were co-evaporated with the molar ratio of FAI:PbI_2= 1:1 on the z-cut quartz substrate in a custom-built thermal evaporator chamber. During the evaporation, the pressure was typically < 5 × 10^{-6} mbar. The sublimation rate of the precursors was controlled using gold-plated quartz microbalances adjacent to the crucible and a PID-loop-control software. Unless specified otherwise, all samples were annealed in an N_2 glovebox at 150 °C for 5 minutes and at 135 °C for 25 minutes. After cooling down to room temperature, films were ready to use.

2.4 $CsPbBr_3$ thin films

A Bridgman-grown single crystal of $CsPbBr_3$ was gently ground into a fine powder in a nitrogen-filled glove box. The starting material was placed in a thermal evaporator crucible. Quartz substrates were ultrasonically cleaned in Hellmanex® III (2% in water), deionized water, acetone, and isopropanol for 15 min at each stage, followed by UV ozone treatment for 10 min. The thickness of the fabricated films was controllable by the mass of the deposition material (200 mg of starting material to obtain 100 nm perovskite film). The vacuum of the evaporation chamber was reduced to 10^{-6} Torr. The substrate temperature was typically 20 °C. The deposition temperature was in the range 400-500 °C. The deposition rate was 0.6 Å/s. The substrate rotation velocity was 10 rpm. After the evaporation, the films were aged at room temperature in a nitrogen-filled glove box for one month.

2.5 $CsPbBr_3$ single crystals

2.5.1 Synthesis and Purification Runs:

6.423 g of CsBr (ChemCraft, 99.999%) and 11.077 g of $PbBr_2$ (Sigma Aldrich, 99.999%) were mixed and ground together thoroughly using a mortar in an Ar glovebox. This material was then flame-sealed under 1.4 x 10^{-2} mbar vacuum into a fused silica ampule (i.d. 10 mm) with a sharp tip. This ampule was placed in the hot zone of a custom-built 3-zone Bridgman furnace

(HTM Reetz), and the temperatures were set to 675 °C, 400 °C, and 400 °C. The sample was left overnight to ensure a full melt and synthesis reaction, then moved through the furnace at a speed of 0.081 mm/min (4.86 mm/hr) while undergoing 0.3 rpm rotation until it had passed outside the furnace. The resulting ingot had some black impurities near the top, so the sample was reset and the same temperature profile applied, and moved through the furnace more slowly (0.042 mm/min, 2.52 mm/hr) to fully segregate these impurities. The resulting ingot was opened in the Ar glovebox and the black regions at the top of the ingot (typically carbon-containing impurities from $PbBr_2$ precursor) were cut off and discarded, and the material was broken into chunks (to reduce the risk of thermal expansion cracking the ampule) and flame-sealed under 1.1 x 10^{-2} mbar vacuum into a new fused silica ampoule (i.d. 10 mm) with a sharp tip. This process removes the impurities for higher-quality growth. A final purification run, with identical conditions to the previous run (0.042 mm/min, 0.3 rpm, temperatures of 675 °C - 400 °C - 400 °C) showed no further black impurities present, confirming that the material was sufficiently pure to yield higher crystallinity.

2.5.2 Bridgman Crystal Growth:

The Vertical Bridgman method was used to grow the large single crystals of $CsPbBr_3$. The ampoule was reset to the hot zone for the Bridgman Growth. The zone 1 temperature was set to 650 °C with a 150°C/hr ramp rate, and held for 12 hours to ensure a full melt before sample motion occurred. The zone 2 and 3 temperatures were set to 375 °C. These temperatures were held for 350 hours while the ampule was moved through the furnace at a rate of 0.015 mm/min (0.9 mm/hr) under 0.3 rpm rotation. After the motion had ceased, the zone 1 temperature ramped to 375 ºC to make the temperature profile in the furnace uniform. The cooling program was set to slow during the phase transitions occurring near 120 and 90 °C, with a 10 °C/hr cooling rate from 375 °C to 175 °C, a 2.5 °C/hr slow cooling rate from 175 °C to 75ºC, and a 10 °C/hr rate to 30 °C. The resulting $CsPbBr_3$ ingot was orange-red and had large (5+ mm) transparent single-crystalline domains, though the edges of some portions exhibited twinning.

2.5.3 Crystal Processing:

The ingot was opened in an Ar glovebox and cut into 2 mm-thick wafers using a Crystal Systems Corporation Cu-02 Desktop Crystal Cutter with Goniometer operating at 60 rpm with oil-based lubricant. The surfaces of these wafers were polished using a Crystal Systems Corporation TP-02 Polisher operating at 20 rpm, with MicroMesh SiC cutting papers used to get successively

finer surfaces with a final polish of 12000 grit producing an optical mirror-like surface. These processing steps were completed under Ar to preserve the pristine surfaces and the crystals were sealed under Ar for transport, ensuring that both the raw material and as-grown crystals were never exposed to ambient conditions.

2.6 PbI_2 thin films

PbI_2 was evaporated with the deposition rate of 0.2Å/s on the z-cut quartz substrate in a custom-built thermal evaporator chamber. During the evaporation, the pressure was typically $< 5 \times 10^{-6}$ mbar. The source rates were kept constant using gold-plated quartz microbalances and a PID-loop-control software.

2.7 $Cs_2AgBiBr_6$ thin films

The stock solution was prepared by dissolving CsBr (Alpha Aesar, 99.999 % metals basis), $BiBr_3$ (Alpha Aesar, 99.9 % metals basis) and AgBr (Alpha Aesar, 99.998 % metals basis) in 1 mL DMSO (Sigma Aldrich, anhydrous, ≥99.9%) by vigorous stirring at 130 °C for 60 minutes to obtain a 0.5 M solution. All Steps were performed in a nitrogen-filled glovebox with controlled atmosphere. The substrates were cleaned with a detergent (Hellmanex), followed by washing with acetone and ethanol and dried under an air stream. Afterwards, the substrates were cleaned with oxygen plasma for 5 minutes and immediately transferred into the glovebox. Prior to the spincoating step, the substrates and the solution were placed on a hotplate (Heidolph with internal temperature sensor) at 60 °C to be preheated. The stock solution was constantly stirred. The thin films were fabricated by spincoating the warm solution dynamically (1000 rpm for 10 s, followed by a second step at 6000 rpm for 35 seconds) onto the preheated substrates (70 µL of the solution were dropped immediately after the substrate started to spin at 1000 rpm). After the spincoating, the thin films were annealed at 275 °C for 5 minutes, and the preheating was set at 60 °C.

2.8 Cu_2AgBiI_6 thin films

Thin films of Cu_2AgBiI_6 were fabricated by vacuum evaporating (BOC Edwards Auto 306) and co-depositing bismuth(III) iodide (Alpha Aesar Puratronic, 99.999%), silver(I) iodide (Alpha Aesar Premion, 99.999%), and copper(I) iodide (Alpha Aesar Puratronic, 99.998%) precursors

from three separate, 2.4 cm^3 alumina crucibles and thermal sources. The crucibles and sources were custom-made by Moorfield Nanotechnology to fit the dimensions of the evaporation chamber. The precursors were heated to the temperature corresponding to the following evaporation rates: CuI, AgI, BiI_3 = 0.33 Ås^{-1} (370°C), 0.18 Ås^{-1} (475°C), and 0.50 Ås^{-1} (230°C), respectively. Cu_2AgBiI_6 films were 250 nm thick ((249 ± 13) nm, as measured by a Veeko Dektak 150 profilometer). The rates were measured using three quartz crystal microbalances (QCM) positioned off centre to each sources' vapour cone and an Inficon SQC-310 deposition controller. Prior to the deposition of Cu_2AgBiI_6, a tooling factor for each precursor was calculated to correct the divergence between the true rate and the rate measured by the QCM. To do this, 100 nm (as measured on the SQC-310 controller) of each precursor was deposited on 30 x 30 mm glass substrates and the thickness was measured using a profilometer. A new tooling factor was calculated using:

$$Tooling\ Factor = Default\ Tooling\ Factor \times Thickness\ (Dektak)\ /\ Thickness\ (QCM)$$

X-ray diffraction was used to ensure only the binary precursors and no other crystalline impurities were deposited. All depositions were carried out under vacuum (~ 2 × 10^{-6} mbar). The substrates were protected during the heating and cooling process by a mechanical shutter, and the substrates were rotated during deposition to improve surface coverage. No intentional substrate heating was applied. However, the substrates reached a maximum temperature of approximately 60°C during co-deposition due to heat transfer from the sources. The temperature of the substrates was measured using RS Electronics PRO non-reversible temperature sensitive labels (RS Stock No.:779-9779).

2.9 AgI thin films

Thin films of silver iodide were fabricated by depositing (using a BOC Edwards Auto 306 evaporator) silver(I) iodide (Alpha Aesar Premion, 99.999%) precursor from 2.4 cm^3 alumina crucibles and thermal sources. The crucibles and sources were custom-made by Moorfield Nanotechnology to fit the dimensions of the evaporation chamber. The precursor was heated to the temperature corresponding to the desired evaporation rates, with the rates measured using a quartz crystal microbalance positioned off centre to the source's vapour cone and an Inficon SQC-310 deposition controller. All depositions were carried out under vacuum (10^{-6} mbar). The substrates were protected during the heating and cooling process by a mechanical shutter, and the substrates were rotated during deposition to improve surface coverage. No

intentional substrate heating was applied. All films are 250-300 nm thick and were deposited on z-cut quartz substrates. Films were annealed post deposition in a nitrogen glovebox for 15 minutes at 180°C. The evaporation rate was 0.2 Ås^{-1}. Films were not annealed post-deposition.

3 Comparison of Raman spectra for AgI and $Cs_2AgBiBr_6$ thin films

For clarity, zoomed-in spectra of AgI and $Cs_2AgBiBr_6$ are plotted below, focusing on the central region close to the elastically scattered light, in order to demonstrate that the central Raman response is visible for AgI Raman spectra but not for $Cs_2AgBiBr_6$. The AgI Raman spectrum clearly exhibits a low-frequency Raman response, whereas $Cs_2AgBiBr_6$ Raman spectrum shows a residual Rayleigh scattering, rather than a response from the material. This difference is also apparent in the reduced spectra shown in Figure 3(a) of the main text, where a slow rise in the Raman response is visible from zero frequencies for AgI but not for $Cs_2AgBiBr_6$.

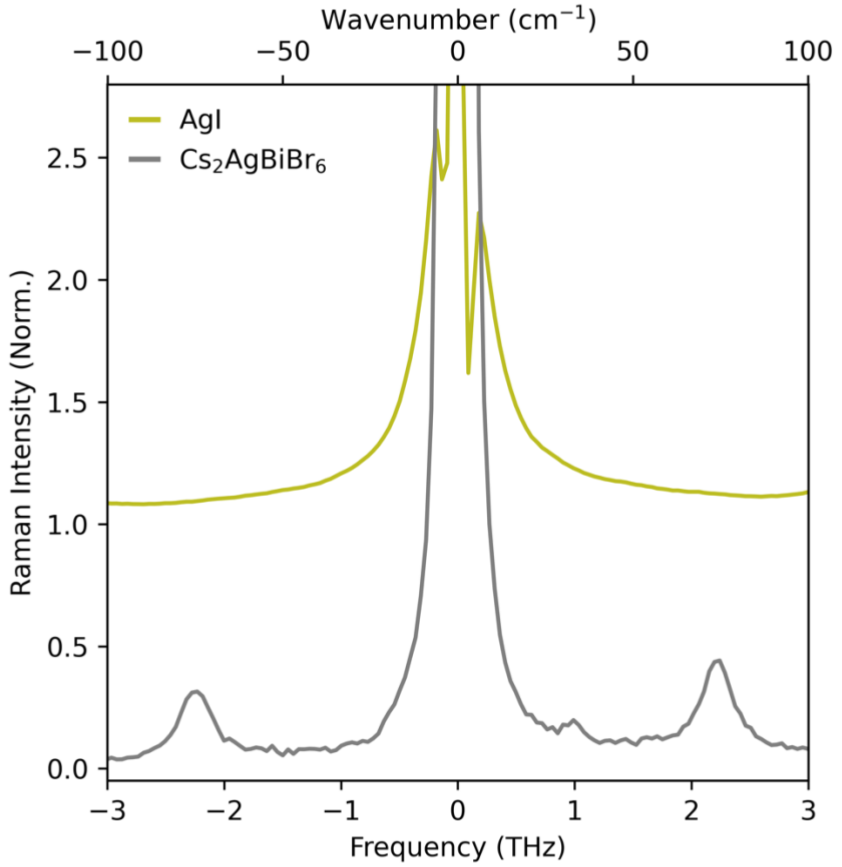

Figure S2. Normalised Raman intensity spectra of AgI and $Cs_2AgBiBr_6$ thin films, focusing on the narrow range close to the central, elastically scattered peak. A CW laser with wavelength 900nm was used as the light source, and the Raman signal was collected in a back-scattering geometry. Rayleigh scatter was suppressed with volume-Bragg notch filters. Experimental details can be found in Supporting Information Section 1 above.

4 Deviation between reduced Raman and IR spectra

Analogous to the example shown for $MAPbI_3$ in Figure 2(d) in the main text, we illustrate here differences between the reduced Raman and reduced IR spectra for the rest of the examined MHPs in the following figure.

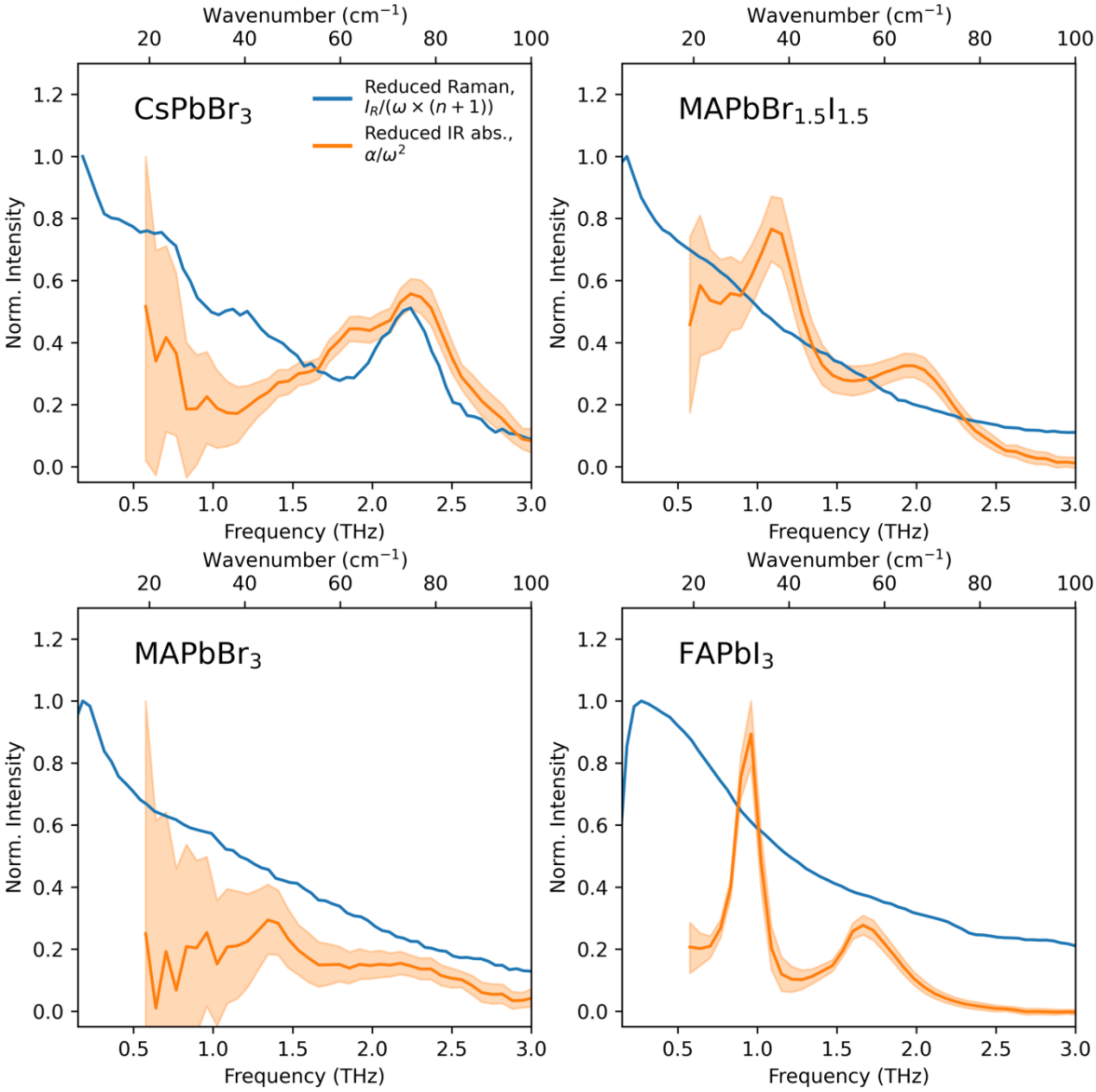

Figure S3. Normalised reduced Raman and reduced IR absorption spectra of $CsPbBr_3$, $MAPbBr_{1.5}Br_{1.5}$, $MAPbBr_3$ and $FAPbI_3$ thin film on z-cut quartz. The reduced Raman spectrum is given by $I_R/(\omega \times (n+1))$, where I_R is the measured Raman intensity, and the reduced IR spectrum is given by α/ω^2, where α is the measured THz IR spectrum. A laser wavelength of 900nm was used for non-resonant Raman spectroscopy and THz-TDS was employed for acquisition of the IR absorption spectrum, as detailed in Supporting Information Section 1 above. The equivalent spectra for $MAPbI_3$ thin films are shown in Figure 2 of the main manuscript.

We also plot Figure 3 in the main text, but with the same frequency axes for (a) and (b).

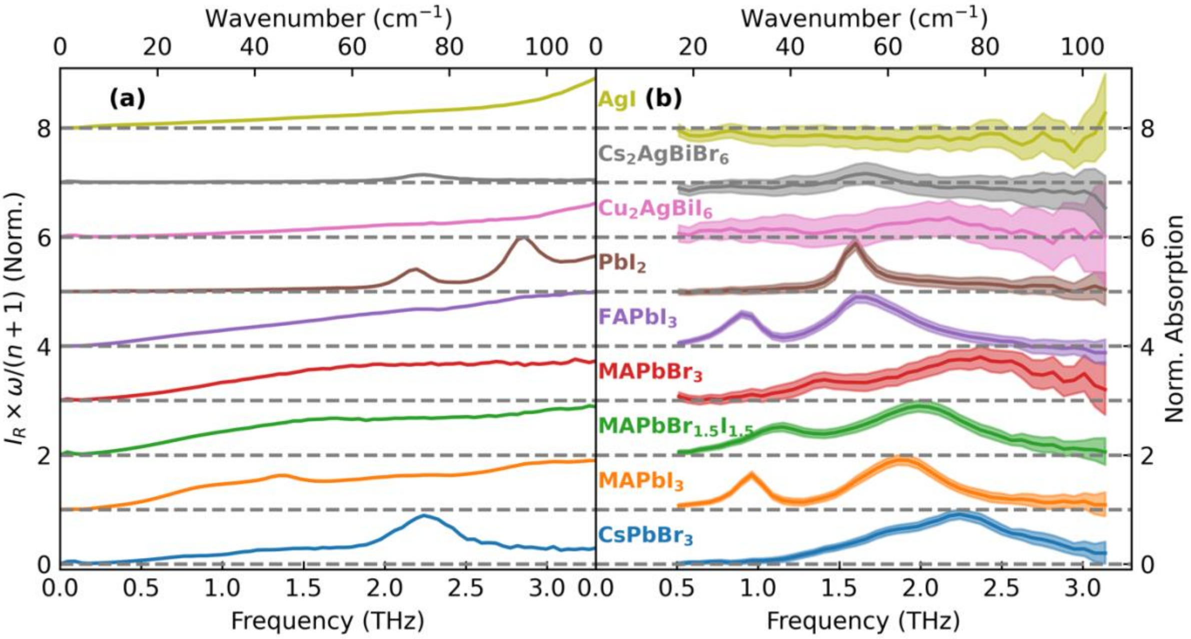

Figure S4 (a) Normalised reduced Raman spectra of metal halides deposited as thin films on z-cut quartz. (b) Normalised IR absorption of the same metal halides (equivalent to the reduced Raman spectra, as can be seen from Equation 1 and 2 of the main manuscript), acquired with THz-TDS.

5 Fitting of Raman and THz IR spectra

The text below describes the method employed for fitting Raman and IR THz spectra of metal halides based on a simple damped harmonic oscillator model, with a table (Table 1) listing the extracted mode frequencies and associated broadening provided at the end.

Raman spectra were fitted with the sum of the individual responses expected for a number of damped harmonic oscillators, with the i-th individual oscillator response given by:

$$S_i^{Raman}(\omega) \; = A_i(n(\omega)+1) \times \mathrm{Im}(1/({\omega_i}^2 - \omega^2 - i\Gamma_\mathrm{i}\omega)),$$

where A_i, n, ω_i, and Γ_i are the amplitude, Bose-Einstein factor, harmonic oscillator frequency, and damping coefficient of i-th mode, respectively.

In addition to these damped harmonic modes, a quasi-elastic scattering response was also added[10] as follows:

$$S_{QE}^{Raman}(\omega) \ = A_{QE}(n(\omega)+1) \times \mathrm{Im}(i\omega/(\gamma_{QE} - i\omega)),$$

where A_{QE} and γ_{QE} are the quasi-elastic scattering amplitude and broadening factor, respectively. We note that from fitting we find that the broadening factor for the quasi-elastic scattering is much smaller (<0.25 THz ~ 8cm^{-1}) than our interested range for comparison with IR spectra; this quasi-elastic response is a different process to what we are interested in in this study. It has been attributed to rattling of molecular cations in a temperature-dependent Brillouin zone scattering study.[10]

Graphs below show the result of such fits to Raman spectra for a range of metal halide semiconductors, with the extracted parameters given in Table 1 below. In some cases, the frequency range of fitting had to be constrained due to either the presence of very strong substrate Raman peaks, or the Raman response being too broad for any effective fitting.

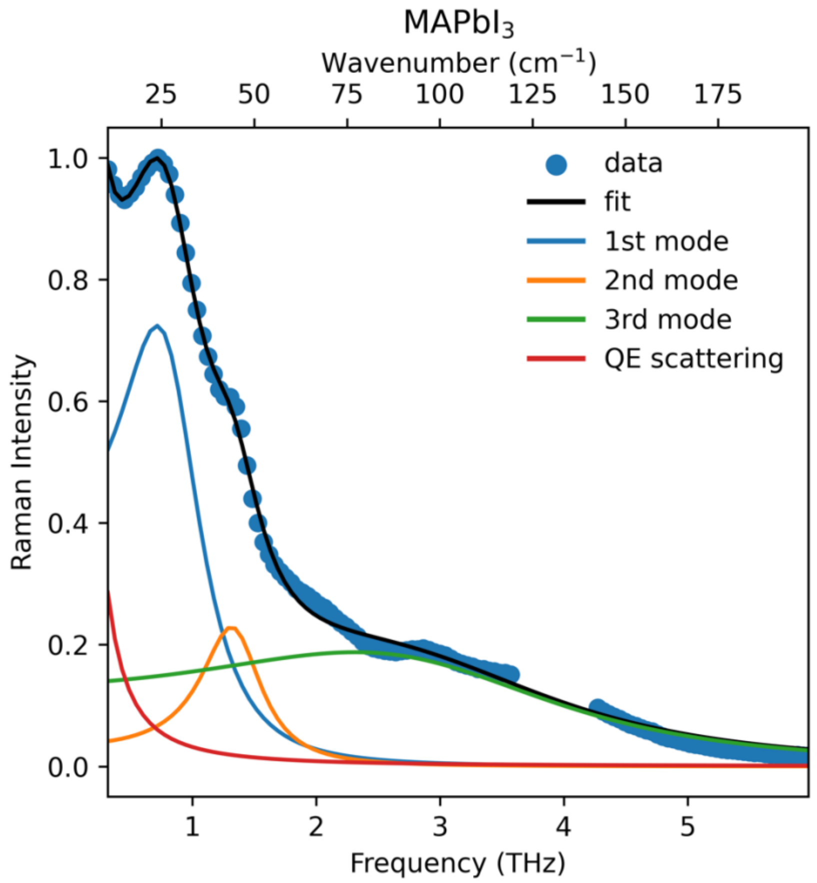

Figure S5. Damped harmonic oscillator model fits (solid lines) to the experimentally recorded (solid circles) Raman response of a $MAPbI_3$ thin film. Fits reflect the sum over three phonon modes, together with a small quasi-elastic (QE) scattering response.

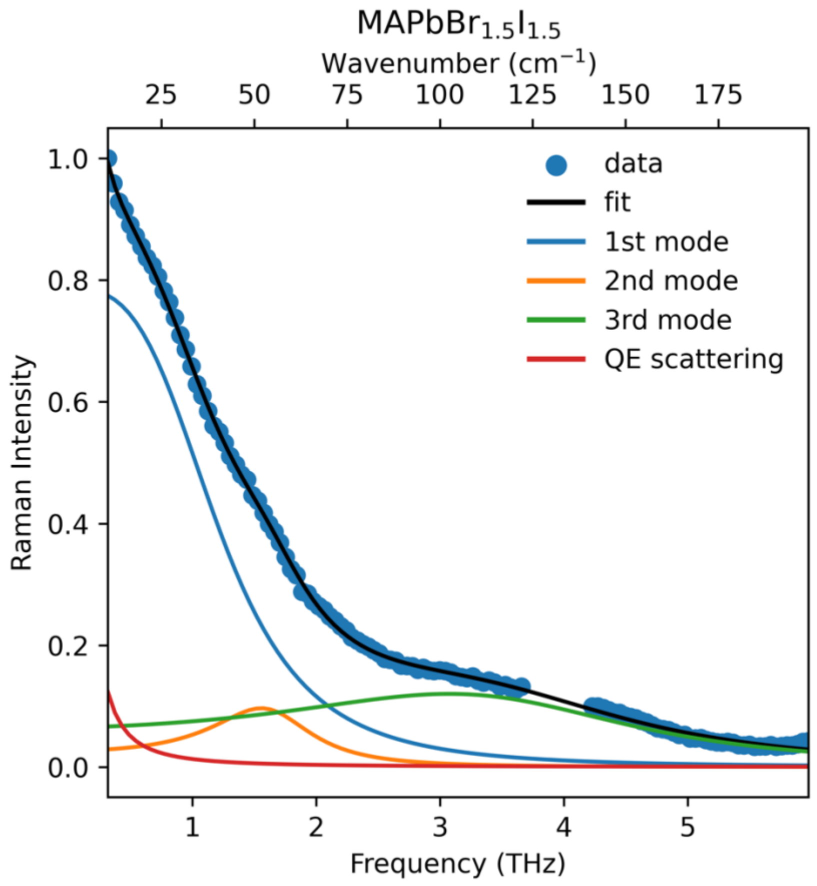

Figure S6. Damped harmonic oscillator model fits (solid lines) to the experimentally recorded (solid circles) Raman response of a $MAPbI_{1.5}Br_{1.5}$ thin film. Fits reflect the sum over three phonon modes, together with a small quasi-elastic (QE) scattering response.

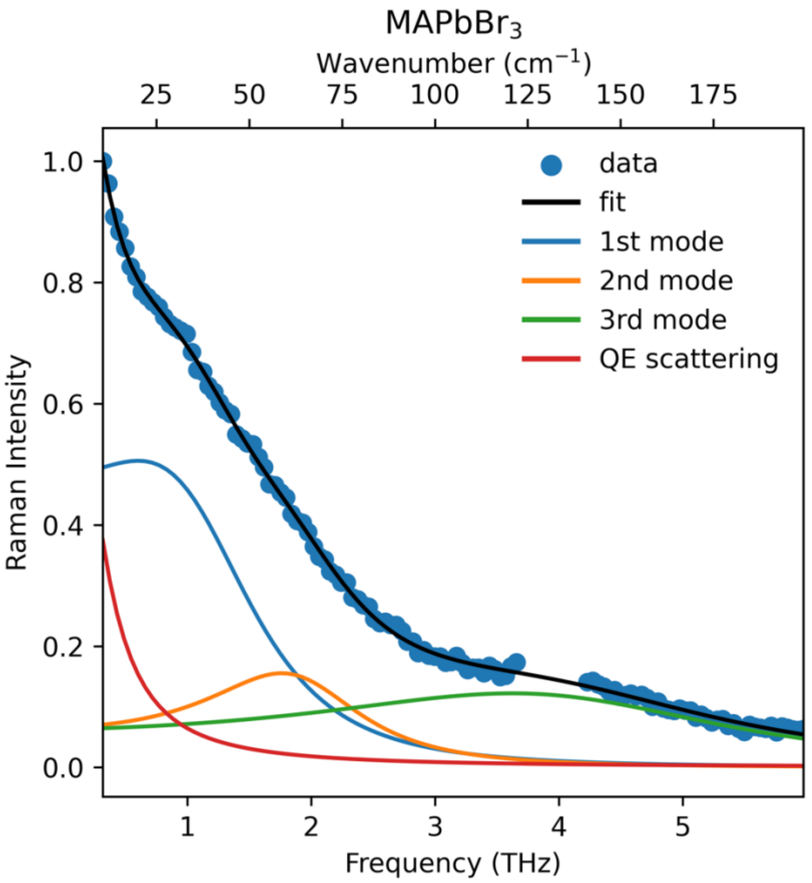

Figure S7. Damped harmonic oscillator model fits (solid lines) to the experimentally recorded (solid circles) Raman response of a $MAPbBr_3$ thin film. Fits reflect the sum over three phonon modes, together with a small quasi-elastic (QE) scattering response.

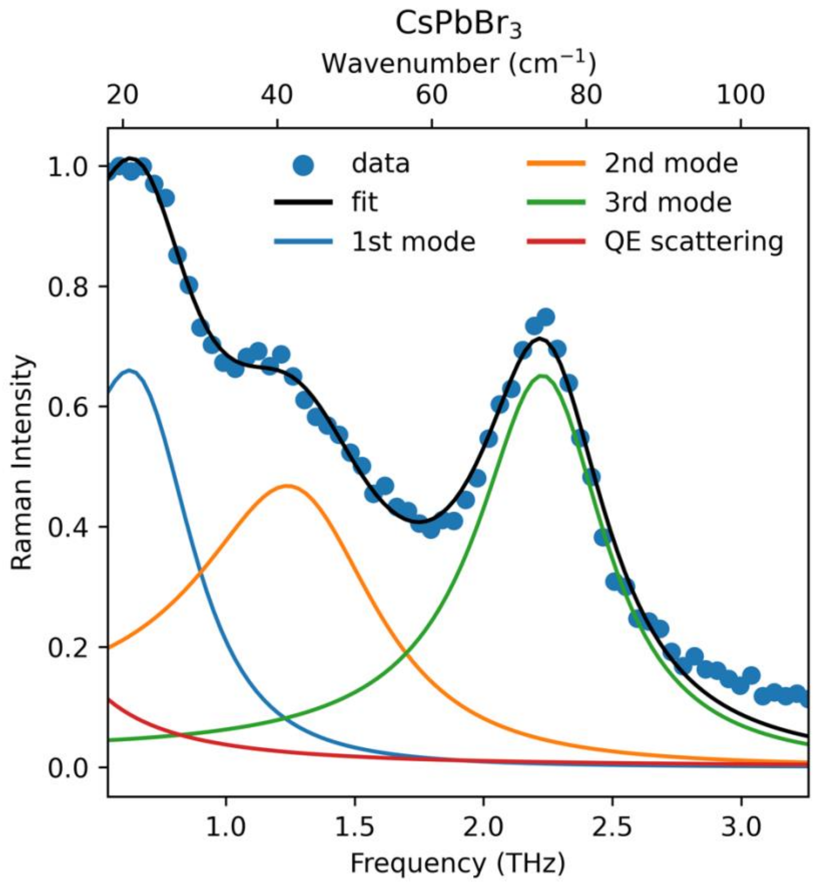

Figure S8. Damped harmonic oscillator model fits (solid lines) to the experimentally recorded (solid circles) Raman response of a $CsPbBr_3$ thin film. Fits reflect the sum over three phonon modes, together with a small quasi-elastic (QE) scattering response.

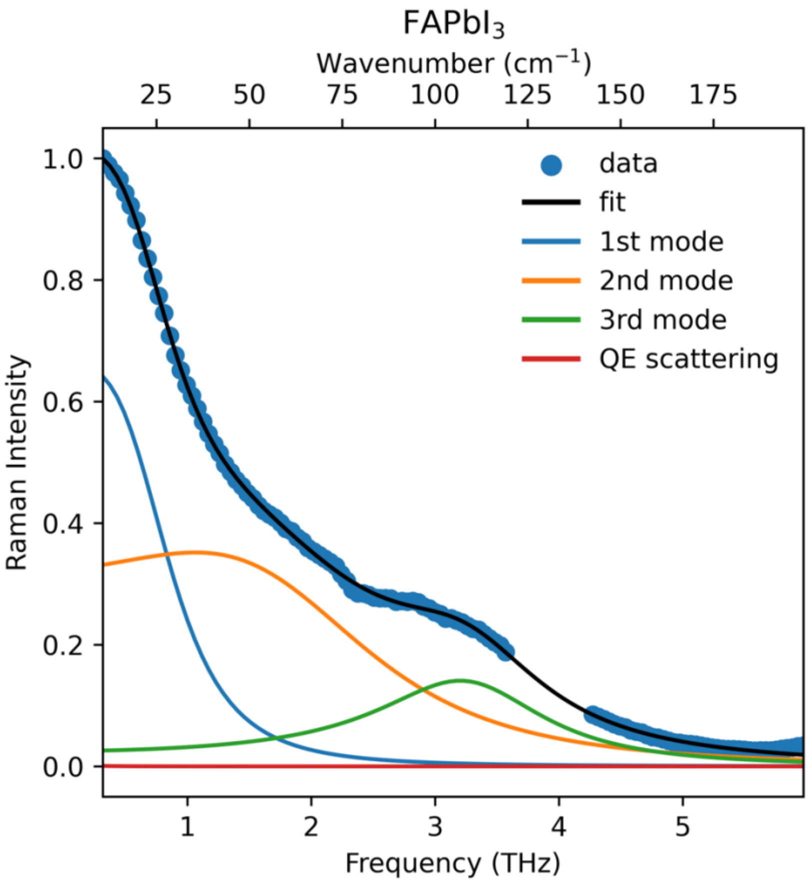

Figure S9. Damped harmonic oscillator model fits (solid lines) to the experimentally recorded (solid circles) Raman response of a $FAPbI_3$ thin film. Fits reflect the sum over three phonon modes, together with a small quasi-elastic (QE) scattering response.

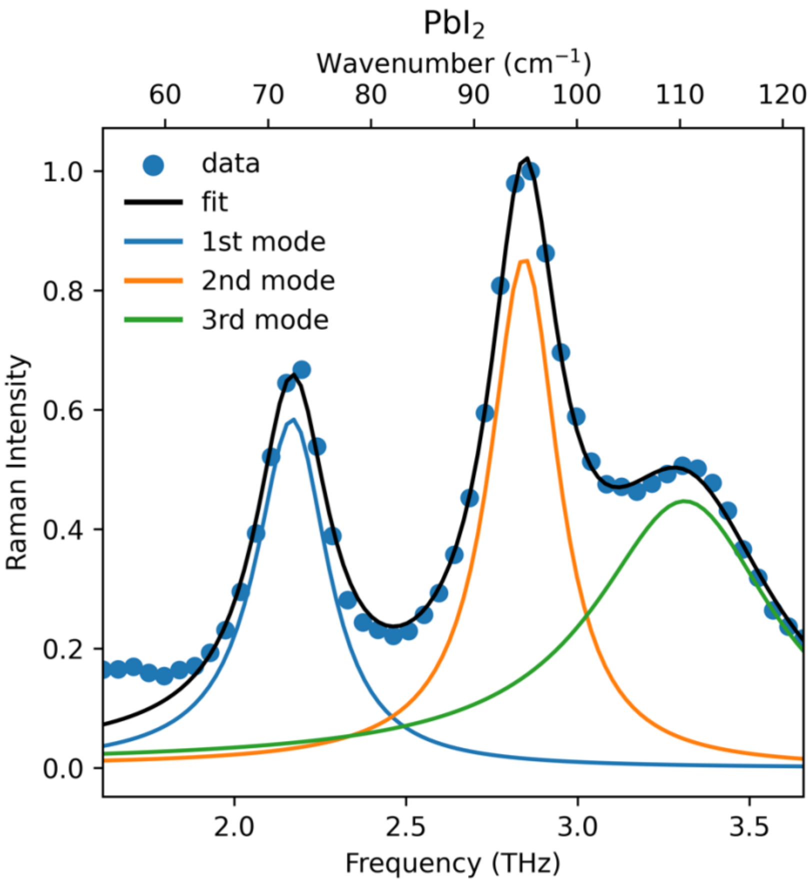

Figure S10. Damped harmonic oscillator model fits (solid lines) to the experimentally recorded (solid circles) Raman response of a PbI_2 thin film. Fits reflect the sum over three phonon modes.

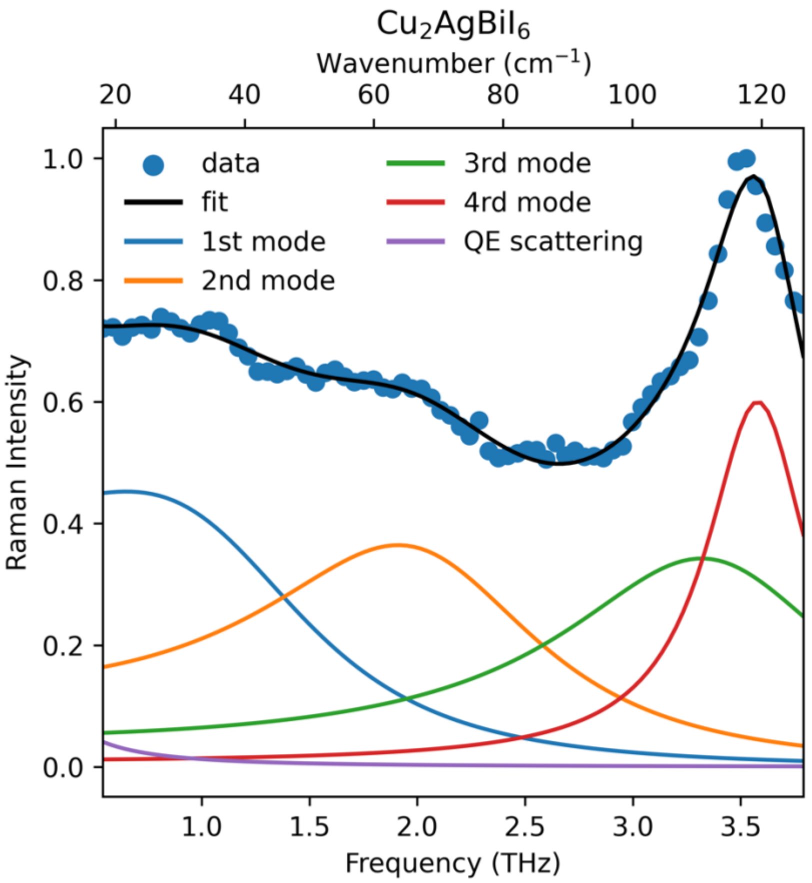

Figure S11. Damped harmonic oscillator model fits (solid lines) to the experimentally recorded (solid circles) Raman response of a Cu_2AgBiI_6 thin film. Fits reflect the sum over four phonon modes, together with a small quasi-elastic (QE) scattering response.

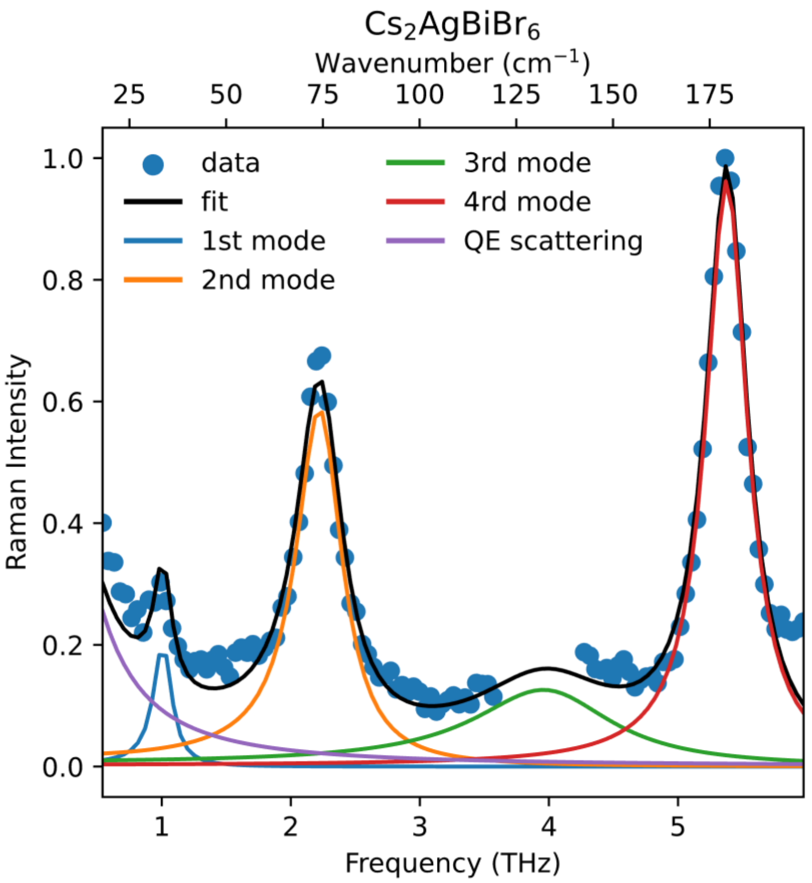

Figure S12. Damped harmonic oscillator model fits (solid lines) to the experimentally recorded (solid circles) Raman response of a $Cs_2AgBiBr_6$ thin film. Fits reflect the sum over four phonon modes, together with a small quasi-elastic (QE) scattering response.

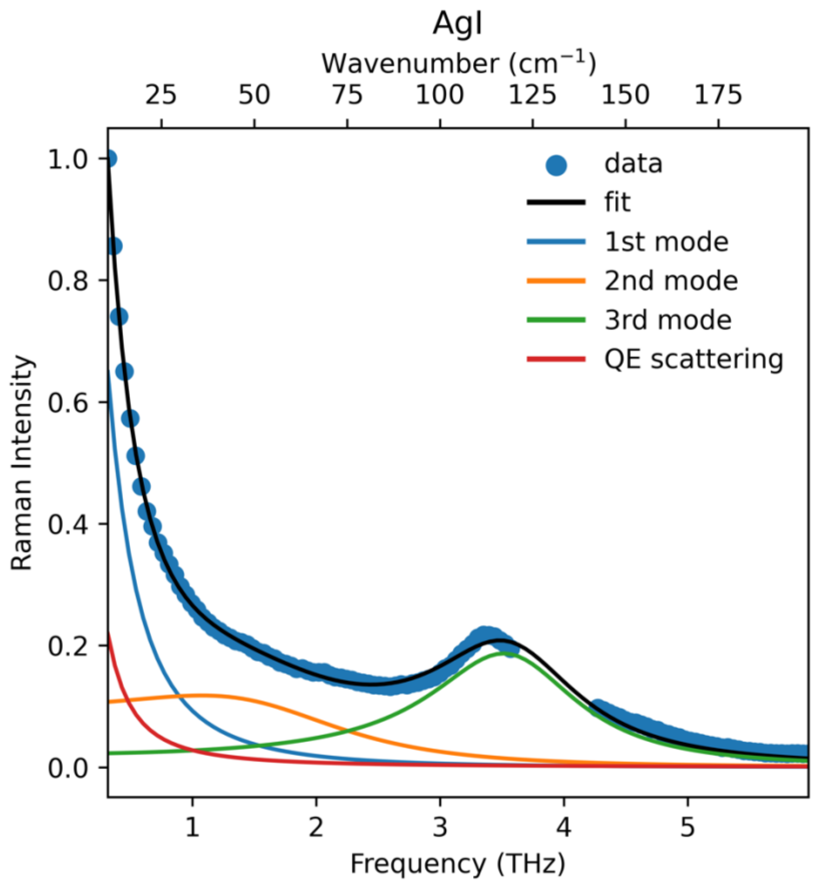

Figure S13. Damped harmonic oscillator model fits (solid lines) to the experimentally recorded (solid circles) Raman response of a AgI thin film. Fits reflect the sum over three phonon modes, together with a small quasi-elastic (QE) scattering response.

For THz spectra, a similar harmonic oscillator response was used for fits to data, however, the $(n(\omega)+1)$ Bose-Einstein term was omitted, given that IR photon absorption and conversion into a phonon does not require prior presence of a phonon population. The response per oscillator mode i is then given by:

$$S_i^{IR}(\omega) \ = A_i \times \mathrm{Im}(1/({\omega_i}^2 - \omega^2 - i\Gamma_\mathrm{i}\omega)).$$

Graphs below show the result of such fits to THz IR spectra for a range of metal halide semiconductors, with the extracted parameters given in Table 1 below.

We also note that because of the focus on the ultra-low range of frequency (<3 THz) in this study, our measurement window may not necessarily capture all of the optically active modes.

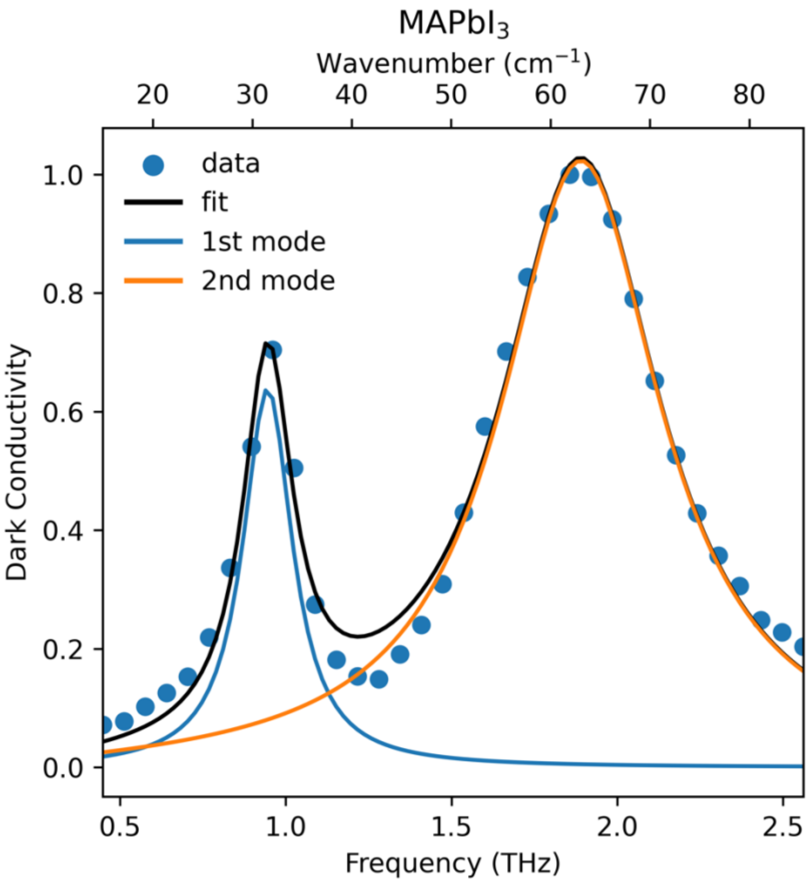

Figure S14. Damped harmonic oscillator model fits (solid lines) to the experimentally recorded (solid circles) IR response in the THz region for a $MAPbI_3$ thin film. Fits reflect the sum over two phonon modes.

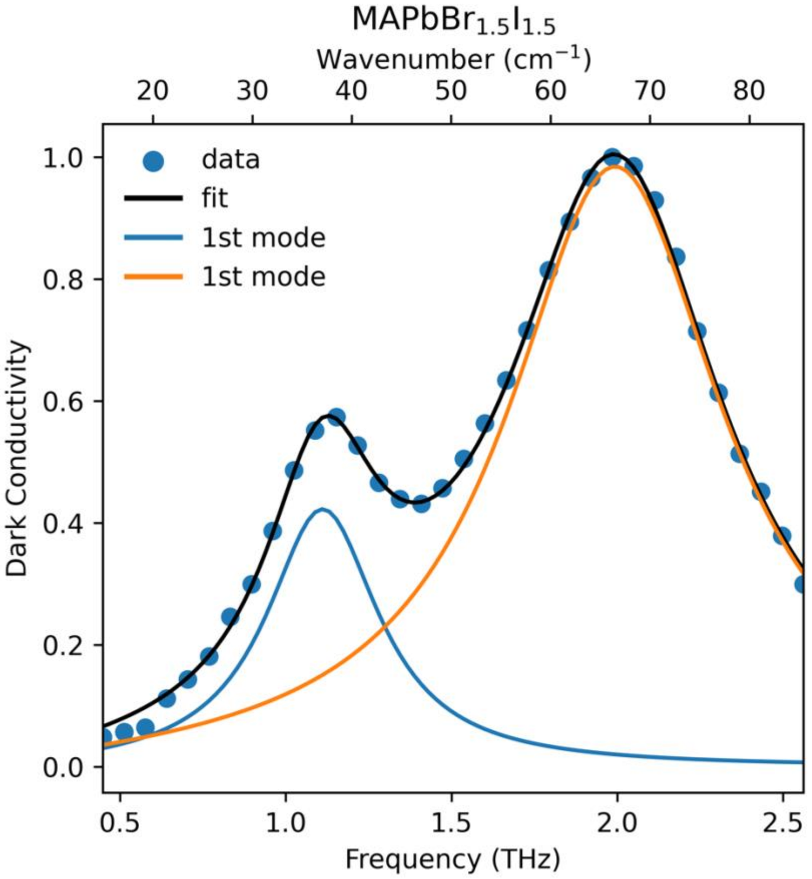

Figure S15. Damped harmonic oscillator model fits (solid lines) to the experimentally recorded (solid circles) IR response in the THz region for a $MAPbI_{1.5}Br_{1.5}$ thin film. Fits reflect the sum over two phonon modes.

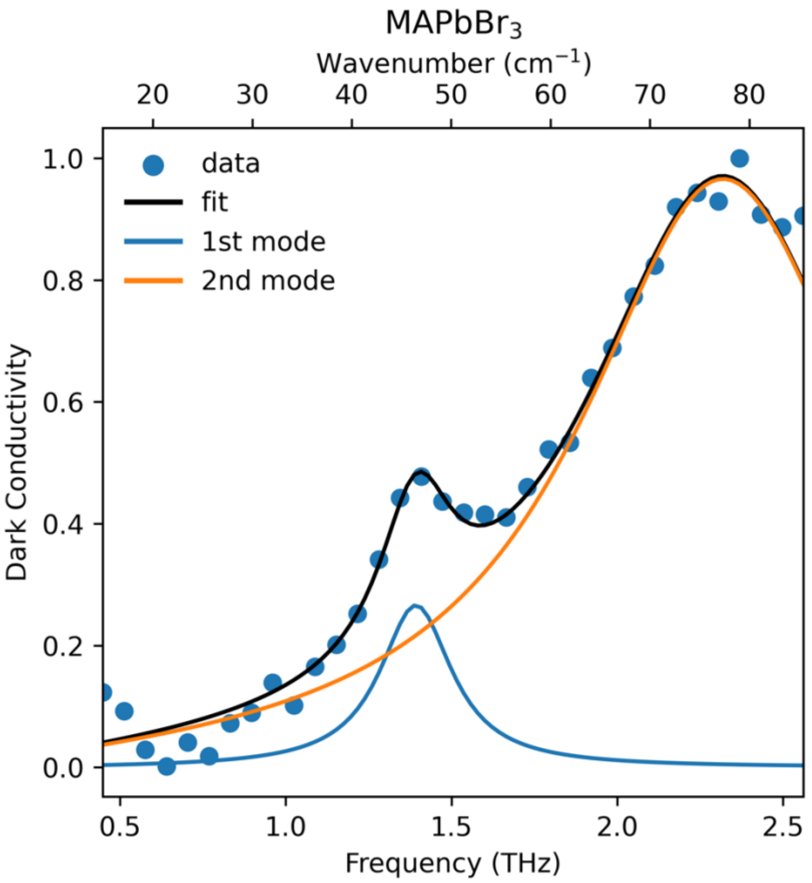

Figure S16. Damped harmonic oscillator model fits (solid lines) to the experimentally recorded (solid circles) IR response in the THz region for a $MAPbBr_3$ thin film. Fits reflect the sum over two phonon modes.

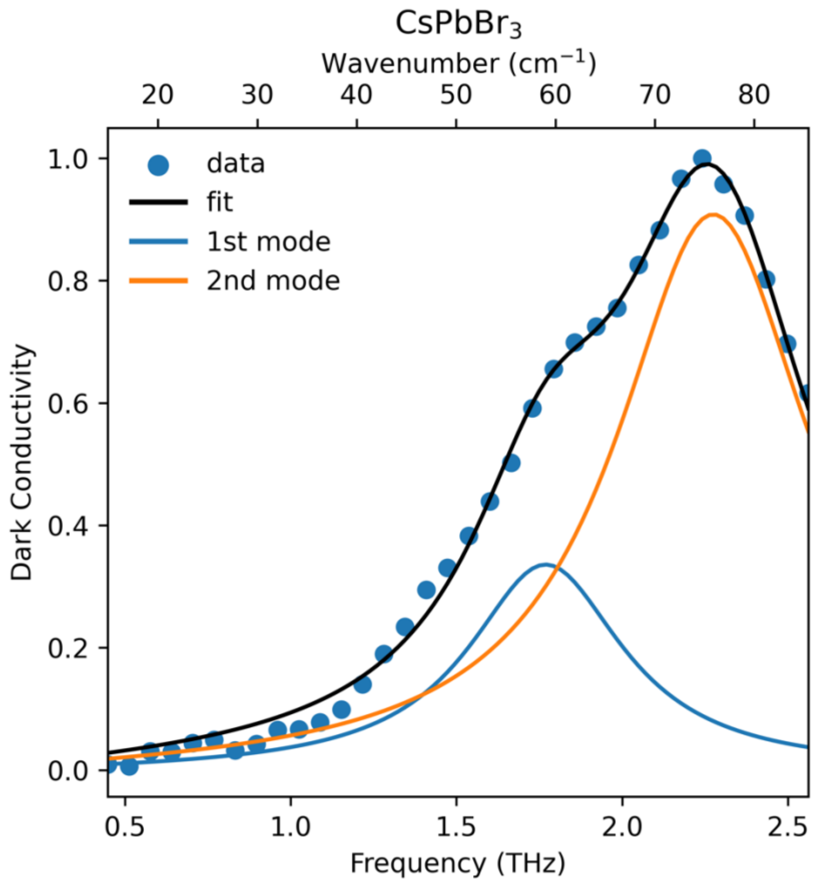

Figure S17. Damped harmonic oscillator model fits (solid lines) to the experimentally recorded (solid circles) IR response in the THz region for a $CsPbBr_3$ thin film. Fits reflect the sum over two phonon modes.

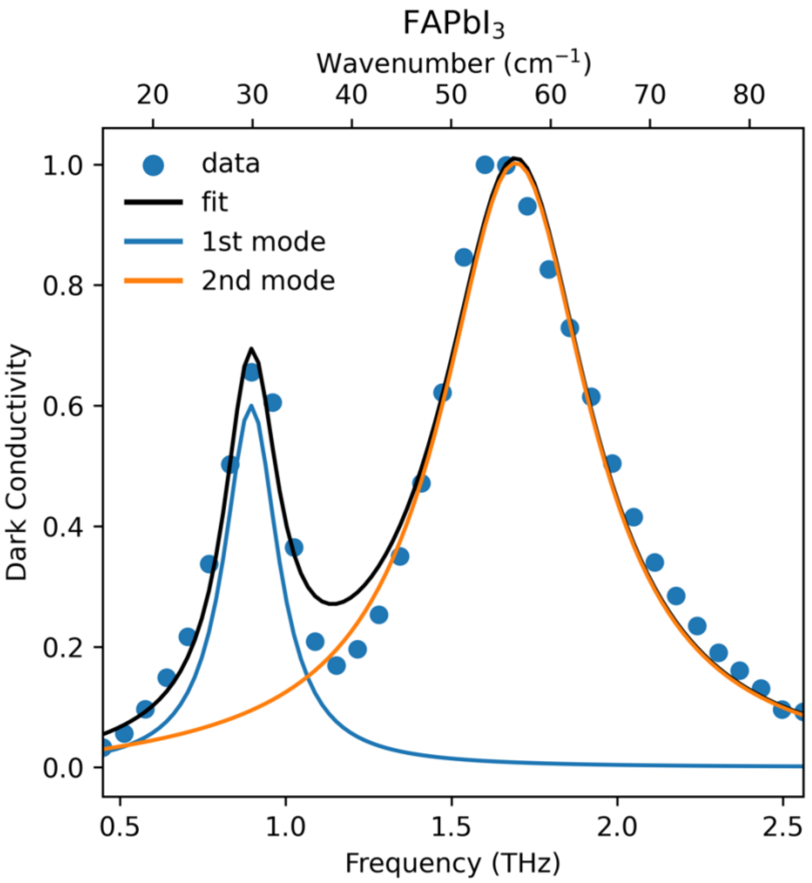

Figure S18. Damped harmonic oscillator model fits (solid lines) to the experimentally recorded (solid circles) IR response in the THz region for a $FAPbI_3$ thin film. Fits reflect the sum over two phonon modes.

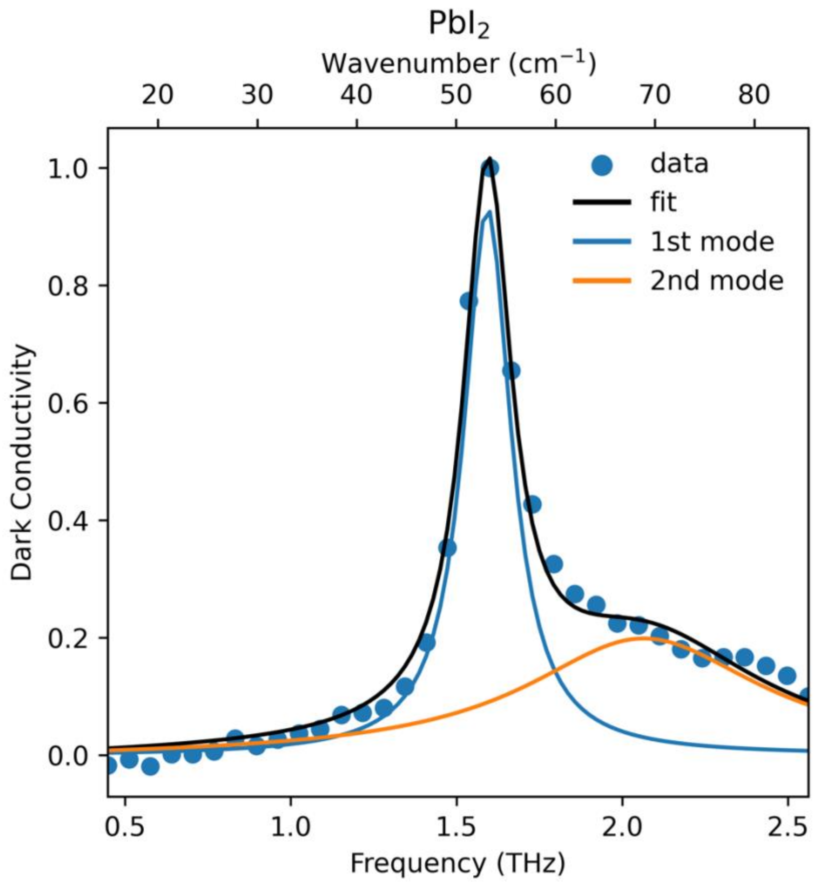

Figure S19. Damped harmonic oscillator model fits (solid lines) to the experimentally recorded (solid circles) IR response in the THz region for a PbI_2 thin film. Fits reflect the sum over two phonon modes.

Material	Raman/IR	Frequency (THz)	Broadening (THz)
$CsPbBr_3$	Raman	0.75	0.59
	Raman	1.37	0.84
	Raman	2.27	0.60
	IR	1.79	0.57
	IR	2.30	0.72
$MAPbI_3$	Raman	0.91	0.82
	Raman	1.38	0.59
	Raman	3.42	3.82
	IR	0.95	0.18
	IR	1.91	0.59
$MAPbBr_{1.5}I_{1.5}$	Raman	1.29	2.03
	Raman	1.70	0.99
	Raman	3.70	3.59
	IR	1.13	0.41
	IR	2.03	0.79
$MAPbBr_3$	Raman	1.43	1.90
	Raman	2.05	1.55
	Raman	4.52	4.17
	IR	1.40	0.27
	IR	2.38	1.04
$FAPbI_3$	Raman	0.88	1.33
	Raman	2.29	3.04
	Raman	3.39	1.66
	IR	0.90	0.20
	IR	1.72	0.55
PbI_2	Raman	2.18	0.25
	Raman	2.85	0.24
	Raman	3.34	0.63
	IR	1.60	0.17

	IR	2.11	0.87
Cu_2AgBiI_6	Raman	1.38	1.78
	Raman	2.19	1.59
	Raman	3.48	1.57
	Raman	3.60	0.57
$Cs_2AgBiBr_6$	Raman	1.01	0.17
	Raman	2.24	0.43
	Raman	4.05	1.31
	Raman	5.38	0.39
AgI	Raman	0.80	2.76
	Raman	2.00	2.49
	Raman	3.64	1.46

Table 1. Oscillator mode frequencies and broadening extracted from fitting damped harmonic oscillator response to Raman and IR spectra in the THz region for various thin films. Fitting details can be found earlier in Section 4.

Graphs below show the extracted oscillator frequency across the $MAPb(Br_xI_{1-x})_3$ thin-film series, which demonstrate the blueshift of all modes with increasing bromide content x in both Raman and IR responses, as would be intuitively expected for increasing incorporation of the lighter halide.

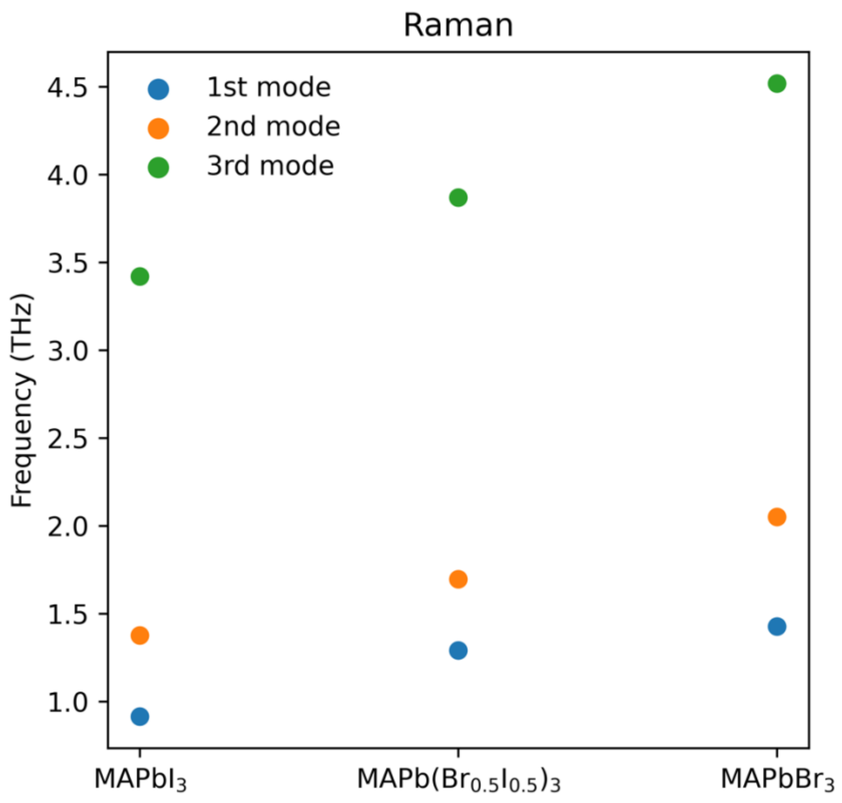

Figure S20. Mode frequencies extracted from fits of the damped harmonic oscillator model to the Raman spectra of $MAPbI_3$, $MAPbI_{1.5}Br_{1.5}$ and $MAPbBr_3$ thin films.

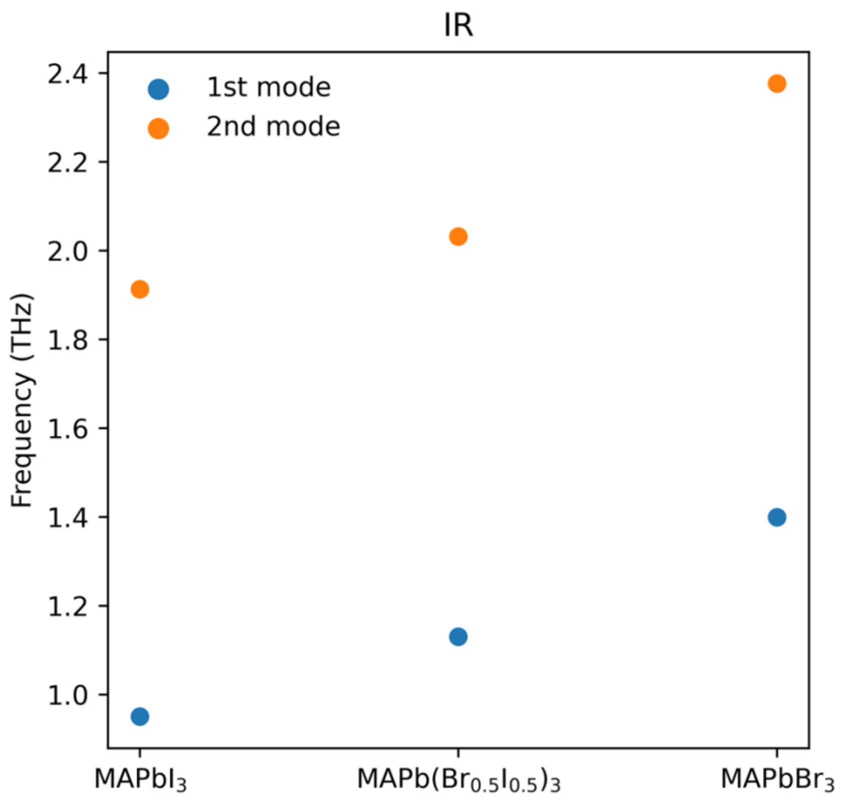

Figure S21. Mode frequencies extracted from fits of the damped harmonic oscillator model to the IR spectra of $MAPbI_3$, $MAPbI_{1.5}Br_{1.5}$ and $MAPbBr_3$ thin films.

References

(1) Glebov, A. L.; Mokhun, O.; Rapaport, A.; Vergnole, S.; Smirnov, V.; Glebov, L. B. Volume Bragg Gratings as Ultra-Narrow and Multiband Optical Filters. In *Micro-Optics 2012*; Proc. SPIE, 2012; Vol. 8428, p 84280C.

(2) Long, D. A. *The Raman Effect: A Unified Treatment of the Theory of Raman Scattering by Molecules*; Wiley, 2002.

(3) Ulatowski, A. M.; Herz, L. M.; Johnston, M. B. Terahertz Conductivity Analysis for Highly Doped Thin-Film Semiconductors. *J. Infrared, Millimeter, Terahertz Waves* **2020**, *41* (12), 1431–1449.

(4) Milot, R. L.; Klug, M. T.; Davies, C. L.; Wang, Z.; Kraus, H.; Snaith, H. J.; Johnston, M. B.; Herz, L. M.; Milot, R. L.; Klug, M. T.; Davies, C. L.; Wang, Z.; Snaith, H. J.; Johnston, M. B.; Herz, L. M.; Kraus, H. The Effects of Doping Density and Temperature on the

Optoelectronic Properties of Formamidinium Tin Triiodide Thin Films. *Adv. Mater.* **2018**, *30* (44), 1804506.

(5) Lim, V. J. Y.; Ulatowski, A. M.; Kamaraki, C.; Klug, M. T.; Miranda Perez, L.; Johnston, M. B.; Herz, L. M. Air-Degradation Mechanisms in Mixed Lead-Tin Halide Perovskites for Solar Cells. *Adv. Energy Mater.* **2023**, *13* (33), 2200847.

(6) Noel, N. K.; Habisreutinger, S. N.; Wenger, B.; Klug, M. T.; Hörantner, M. T.; Johnston, M. B.; Nicholas, R. J.; Moore, D. T.; Snaith, H. J. A Low Viscosity, Low Boiling Point, Clean Solvent System for the Rapid Crystallisation of Highly Specular Perovskite Films. *Energy Environ. Sci.* **2017**, *10* (1), 145–152.

(7) Knight, A. J.; Patel, J. B.; Snaith, H. J.; Johnston, M. B.; Herz, L. M. Trap States, Electric Fields, and Phase Segregation in Mixed-Halide Perovskite Photovoltaic Devices. *Adv. Energy Mater.* **2020**, *10* (9), 1903488.

(8) Lin, Q.; Armin, A.; Burn, P. L.; Meredith, P. Near Infrared Photodetectors Based on Sub-Gap Absorption in Organohalide Perovskite Single Crystals. *Laser Photon. Rev.* **2016**, *10* (6), 1047–1053.

(9) Saidaminov, M. I.; Abdelhady, A. L.; Murali, B.; Alarousu, E.; Burlakov, V. M.; Peng, W.; Dursun, I.; Wang, L.; He, Y.; MacUlan, G.; Goriely, A.; Wu, T.; Mohammed, O. F.; Bakr, O. M. High-Quality Bulk Hybrid Perovskite Single Crystals within Minutes by Inverse Temperature Crystallization. *Nat. Commun.* **2015**, *6*, 7586.

(10) Hehlen, B.; Bourges, P.; Rufflé, B.; Clément, S.; Vialla, R.; Ferreira, A. C.; Ecolivet, C.; Paofai, S.; Cordier, S.; Katan, C.; Létoublon, A.; Even, J. Pseudospin-Phonon Pretransitional Dynamics in Lead Halide Hybrid Perovskites. *Phys. Rev. B* **2022**, *105* (2), 024306.
